# Advanced AI approaches for the modeling and optimization of microgrid energy systems

**DOI:** 10.1038/s41598-025-96145-w

**Published:** 2025-04-12

**Authors:** Mohammed Amine Hoummadi, Badre Bossoufi, Mohammed Karim, Ahmed Althobaiti, Thamer A. H. Alghamdi, Mohammed Alenezi

**Affiliations:** 1 LIMAS Laboratory, Faculty of Sciences Dhar El Mahraz, Sidi Mohammed Ben Abdellah University, Fez, 30003 Morocco; 2 Department of Electrical Engineering, College of Engineering, Taif University, P.O. Box 11099, Taif City, 21974 Saudi Arabia; 3 Wolfson Centre for Magnetics, School of Engineering, Cardiff University, Cardiff, CF24 3AA UK; 4 Electrical Engineering Department, Faculty of Engineering, Al-Baha University, 65779 Al-Baha, Saudi Arabia

**Keywords:** Energy optimization, Renewable integration, Algorithmic energy management, Microgrid, Turbine, Energy science and technology, Renewable energy

## Abstract

The present study examines AI techniques to reduce the cost and CO_2_ emissions for designing and controlling microgrid at minimum cost and providing a power supply to a residential complex of 100 units. Three AI techniques, Genetic Algorithm (GA), Artificial Bee Colony (ABC), and Ant Colony Optimization (ACO), are employed to optimize the optimal composition of energy sources based on solar energy and wind energy, battery storage, and load profiles. GA, from natural selection, is constantly seeking the best configuration. ABC models honeybee foraging behavior to achieve efficient exploration, and ACO models ant colony decision-making to achieve optimal energy configuration. These AI models maximize the use of renewable energy, reduce wastage, and improve microgrid resilience and responsiveness to supply and demand fluctuations. Experiments demonstrate the revolutionary potential of AI to control microgrids. The optimization achieves the lowest electricity cost of 0.037 USD/kWh, a reduction by 67% from Fez’s reference cost (0.115 USD/kWh) and guarantees a supply of power. These results illustrate the ability of AI to power cheap and clean energy systems.

## Introduction

Recently, renewable-based hybrid energy systems have grown in popularity. It is mostly due to rising environmental concerns, increased consumption of energy, increased fuel costs, and the depletion of fossil fuels. Wind and solar energy technologies, in particular, have become more environmentally friendly. Leading to alternatives for ethical and sustainable electricity delivery in rural or off-grid regions. Solar photovoltaic (PV) energy conversion systems with storage^1^ have shown to be an appealing choice for delivering power to rural or off-grid places^2^, Residential dwellings^3,4^, off-grid locations^5,6^, and business structures. On the other hand, the efficiency of PV generation is small, and the power cost per kWh is huge. Consequently, the amount of wind-based power generated significantly increased. Numerous research has focused on the feasibility and optimal size of wind-based systems^7–9^. However, the fundamental drawback of both wind and solar energy is their stochastic character, which creates concerns regarding the user’s accessibility to electrical power. As a result, combining solar and wind energy is a viable option for increasing reliability. Even if it complicates the system, one’s flaws can be compensated by another’s strengths^10^.

Moreover, several studies examined the cost-effectiveness of standalone solar wind hybrid energy systems. The necessity for backup power sources is a fundamental drawback of a standalone solar wind-based energy system due to the irregular nature of both wind and solar resources. A diesel generator or energy storage equipment (e.g. batteries or ultra-capacitors) is often used as a backup when using a stand-alone hybrid system. Its usage raises cost and environmental concerns. However, as technology advances, multiple renewable energy sources are integrated alongside wind and solar power, namely biomass, biogas, micro-hydro, and fuel cells.

Among the aforementioned renewable energy sources, biomass looks to be a more viable option, particularly in countries with a significant agricultural economy. Biomass may be used to produce a wide range of goods, including heat, electricity, and biofuels. Consequently, Energy created by biomass gasifiers is gaining popularity in rural regions. Biomass-powered power plants have a high load factor and are cost-effective^10^. Stand-alone and grid-connected PV biomass with or without storage are viewed as a realistic and inexpensive source of power, particularly in developing countries. Consequently, adopting accessible local renewable energy sources in areas without grid connectivity or with limited power generation can be feasible. Therefore, the intelligent usage of these resources can help satisfy the electrical needs of these areas.

In addition, the storage of the energy created by a hybrid renewable energy system requires a big battery bank. In this scenario, the creation of a conventional self-sustaining hybrid energy system necessitates applying renewable energy sources and batteries. Numerous considerations, including the system’s overall cost, quantity, and renewable energy sources capacity, are critical for hybrid systems. Due to the intermittent nature of renewable energy sources, a hybrid system must have the best possible power flow across all of its components. In hybrid systems, the reliability and the cost of generating energy are the most important aspects. The finest elements should be employed in a well-designed system to ensure reliability.

Given the lack of literature on PV, wind, and biomass-based hybrid systems with energy storage. The authors used AI methods to carry out the economic analysis, and Rehman et al.^11^ suggested the components selection for a hybrid system based on wind and PV for a site in Fez. A mixed integer linear programming-based approach has been created to build hybrid systems. Garrido et al.^12^ also used AI methods to present a techno-economic analysis of a hybrid PV-biomass energy system for a remote area in Fez. The findings suggest that wastes from agriculture and food processing may be important in producing energy, especially in rural areas.

The aforementioned literature demonstrates that for performance analysis, researchers have either used software tools or traditional optimization techniques. However, compared to current optimization techniques, software tools have shown significant drawbacks (e.g. single-function minimization, black box coding, and a longer computation time). In addition, numerous studies on hybrid systems suggested various traditional and evolutionary strategies to obtain the appropriate component size for hybrid systems.

Traditional methods like the graphical construction approach^13^, iterative technique^14^, trade-off method^14^, and linear programming^14^ have been used to complete various research projects. However, these methods might suffer limitations in local minima. To address these shortcomings and ensure the best sizing of hybrid energy systems, researchers commonly used meta-heuristic evolutionary algorithms including the ant and bee colony algorithm^15^, the genetic algorithm^15^, particle swarm optimization^16^, harmony search^17^, and bio-geography-based optimization^17^.

To the extent of our knowledge, a few studies examined the means to optimize a hybrid PV-wind-biomass system and an energy storage system. According to the research, a hybrid system comprised of PV, wind, and biomass, as well as an energy storage system is necessary in remote or off-grid settings. Any hybrid system’s equipment sizing requires difficult labor. Regardless of material from existing studies, the suggested attempt focuses on a hybrid energy system, which integrates solar, wind, biomass, and energy storage.

The aforementioned hybrid systems’ entire ideal component sizing has been determined using either software tools or both traditional and evolutionary approaches. However, none of the studies has examined the use of evolutionary algorithms to determine the best size for PV-wind-biomass systems with battery banks serving as storage. The combination of biomass resources with wind and solar energy can increase the hybrid system’s dependability. Thus, this study proposes an autonomous hybrid PV-wind-biomass system with batteries to meet the electricity needs of a typical hamlet.

The artificial bee colony (ABC) meta-heuristic method has been used to achieve the best configurations for the suggested system. The primary way that the ABC algorithm differs from other algorithms (like GA and PSO) is that it uses fewer control parameters. Like other evolutionary algorithms, it also has good convergence accuracy and the ability to provide optimal outcomes^18^. The findings gathered by the ABC algorithm^18^ have been compared with GA and Ants colonization algorithm to assess how well the implemented technique performed. Using the levelized cost of energy (LCOE), a quick comparison is made. The ideal arrangement is regarded as having the lowest LCOE. These are the primary goals of this effort, in brief. To create a mathematical model of a self-sufficient PV^19^, wind, and biomass energy system with a battery bank to supply electricity to a remote site. Using a swarm-based ABC method, the net present cost (NPC) of the proposed system will be minimized to determine the ideal size of the components with the lowest LCOE to evaluate results from the ABC algorithm against outcomes from GA and Ants colony algorithm and monitor the hybrid system’s performance^20^ in extreme situations like the failure of any generating unit.

The shape of a dependable, reasonably priced hybrid PV-wind-biomass energy system with battery storage that meets the electrical load demand of a small area with an abundance of natural resources is the primary contribution of this study. We went over the operational strategy and mathematical modeling of key system components in detail. We performed a rigorous cost study of the suggested hybrid system using three evolutionary approaches. Finally, we examined the outcomes of several techniques for appropriate size and timing.

In contrast to previous studies focusing solely on conventional optimization methods, this research explores the innovative application of AI techniques—Genetic Algorithm (GA), Ant Colony Optimization (ACO) algorithm, and Artificial Bee Colony (ABC) algorithm—for microgrid optimization.

## Literature review

In the realm of energy microgrid management, numerous studies have delved into exploring sustainable energy sources and employing various optimization techniques to ensure efficient and reliable power supply. A comprehensive survey by^1–3^ titled “Survey of Sustainable Energy Sources for Microgrid Energy Management: A Review” serves as a foundational exploration of classical methods, metaheuristic, and emerging artificial intelligence (AI) techniques for energy microgrid management.

The survey meticulously dissects the strengths and limitations of classical methods, shedding light on their historical significance in addressing microgrid challenges. Additionally, it elucidates the application of metaheuristic approaches, providing insights into their adaptability and efficacy in optimizing energy distribution within microgrid systems. Furthermore, the survey navigates through the landscape of cutting-edge AI methodologies, elucidating how strategies such as Genetic Algorithms (GA), Ant Colony Optimization (ANTS), and Artificial Bee Colony (ABC) algorithms are reshaping the microgrid energy management paradigm.

Building upon this foundational survey, the current work advances the field by introducing a comprehensive mathematical model for microgrid systems. The research extends beyond theoretical constructs, engaging in practical dimensioning exercises aimed at meeting the energy demands of a substantial residential community. In particular, the authors undertake the ambitious task of providing electricity to 100 houses, each with an average power requirement of 47 kW.

The formulation of the microgrid optimization problem is a notable contribution, incorporating not only the electricity demand constraints but also integrating environmental and economic considerations. This multi-dimensional problem is tackled using state-of-the-art AI methods implemented in Python, namely GA, ANTS, and ABC algorithms. The application of these algorithms represents a significant stride towards achieving an optimal microgrid configuration that balances the intricate interplay of energy needs, environmental impact, and economic feasibility.

To gauge the effectiveness of the proposed AI-driven approach, the study conducts a thorough comparative analysis with results obtained from the HOMER (Hybrid Optimization of Multiple Energy Resources) software. This benchmarking exercise adds a layer of credibility to the findings, showcasing the real-world applicability and robustness of the developed optimization framework.

In summary, this work stands as a pivotal contribution to the field of microgrid energy management. By seamlessly integrating theoretical underpinnings with practical dimensioning and advanced AI optimization techniques, the authors not only advance the academic discourse but also provide a tangible framework for addressing the complex challenges associated with sustainable and efficient microgrid energy management.

We conducted a comparative analysis using the HOMER software to assess the performance of our model against industry standards. The results of these comparisons confirm the superiority of our AI-driven optimization approach in achieving optimal energy resource allocation and cost reduction within the microgrid system.

Recent studies have made significant contributions to the field of microgrid energy management and control. For instance, a systematic review of energy management systems based on adaptive controllers with optimization algorithms has provided a comprehensive framework for optimizing microgrid performance. Additionally, the application of perturb and observe (P&O) algorithms for maximum power point tracking (MPPT) in grid-connected PV systems has demonstrated effective reduction in total harmonic distortion (THD). Advanced control strategies, such as PI and fuzzy logic controllers combined with the slime mound algorithm (SMA), have been employed for battery energy management and power control in microgrids. Furthermore, studies on feed-in tariffs and inertia compliance in IEEE bus systems have highlighted the importance of sensitivity analysis and energy management in grid-connected renewable energy systems. Lastly, real-time optimization of battery management in hybrid PV and wind turbine (WT) microgrids using modified slime mound algorithms (MSMA) and fuzzy-PID controllers has shown promising results in achieving optimal performance. These advancements underscore the critical role of AI-driven and optimization-based approaches in enhancing the efficiency, resilience, and cost-effectiveness of modern microgrid systems.

Other studies have attempted to develop a variety of AI-based optimization techniques in microgrid energy management. As an instance, have used a hybrid GA-PSO approach for renewable energy integration optimization and obtained an important reduction in the cost of operation. In another instance, used Artificial Bee Colony (ABC) algorithms to maximize the utilization of energy storage in off-grid microgrids and achieved a 30% efficiency improvement also used ACO to maximize real-time energy dispatch in smart grids by 25% reduction of CO_2_ emissions. Despite referencing AI deployment achievement in microgrid management, the analyses of such work lack an appraisal of their performing detailed evaluation across various schemes of optimization. Contrary to earlier studies, the current piece compares GA, ABC, and ACO altogether and proves the efficiency of applying these algorithms towards establishing cost-effective and sustainable microgrids. With the combination of these methods, our research facilitates the development of intelligent, low-cost, and low-emission energy systems for residential communities.

## Microgrid model

### Microgrid components

**Fig. 1:**
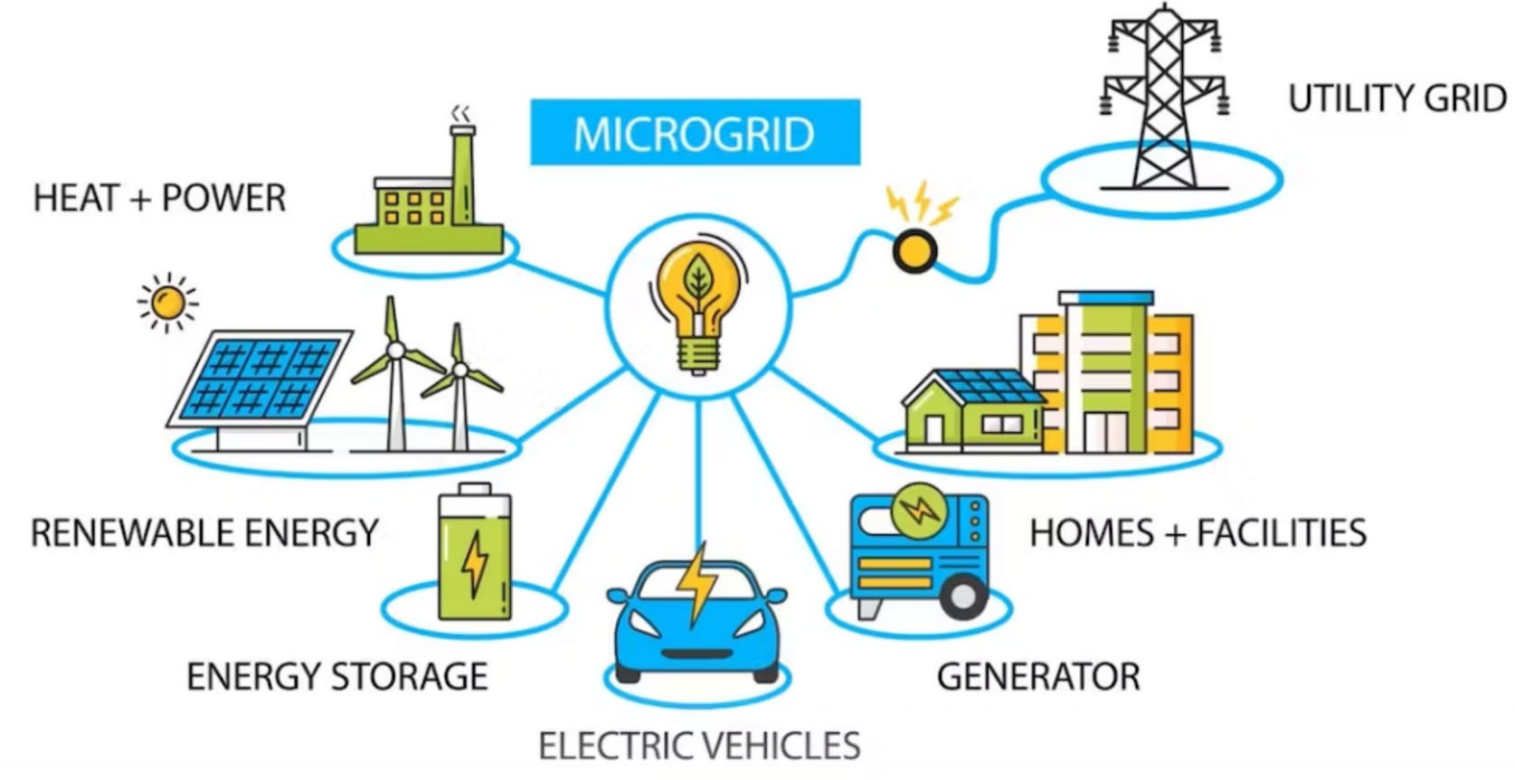
Microgrid components.

An energy system that integrates several power generating, energy storage, and distribution technologies is known as a microgrid. It is a localized, small-scale, and decentralized energy system^21^. It serves a particular geographic area, such as a neighborhood, building complex, university campus, or military base, and works independently of or in conjunction with the primary power grid. The ability to produce power from renewable energy sources (such as solar panels and wind turbines) and conventional sources (such as diesel generators), store extra energy for later use, and efficiently control energy consumption^21^ are some of a microgrid’s important characteristics. To increase energy resilience, lower carbon emissions, increase energy efficiency, and give communities more control over their energy supply and demand, microgrids were developed. This is especially true in times of grid outages^22^ or other emergencies (Fig. 1).

### Wind turbine

The power output of the turbine is detailed by the formula (1) as follow:

1 $$Pm=cp(\lambda,\beta)\:(\mathcal{P}*A/2)\:v^3$$

**Fig. 2:**
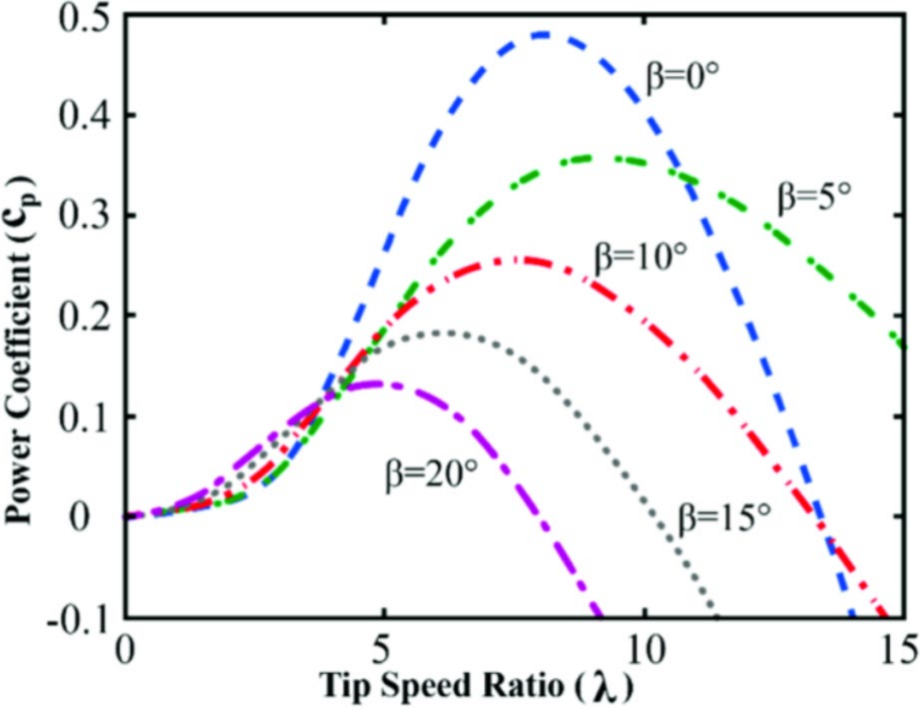
Cp variation.

**Fig. 3:**
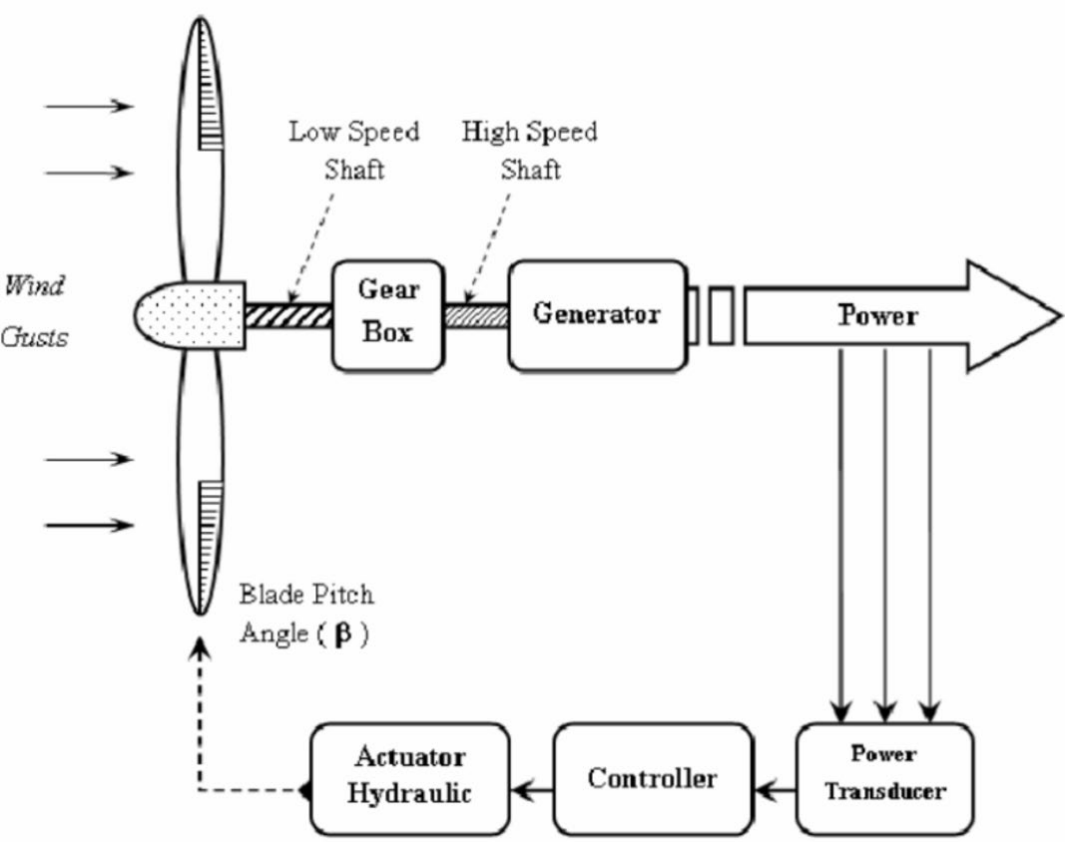
wind turbine model.

**Fig. 4:**
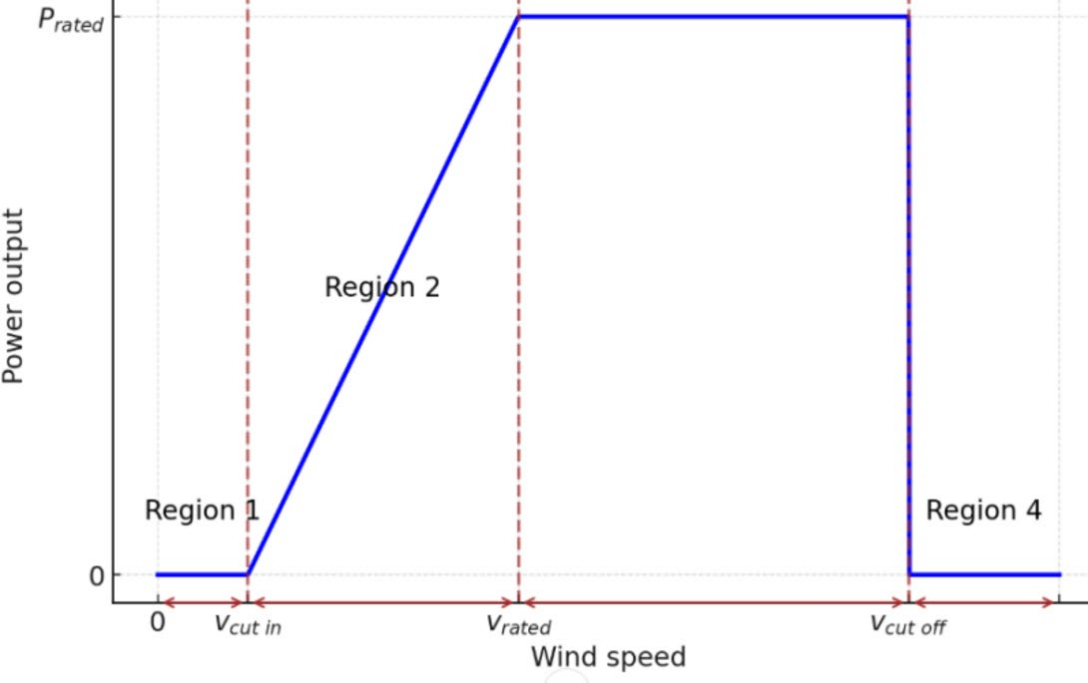
wind turbine output power.

For Cp the turbine performance coefficient, we have (Fig. 2):

The optimum value is obtained for (8.1, 0°), the turbine needs to be in front of the air flux to have the maximum Cp. A detailed view can be explained as follows (Fig. 3):

We can explain the wind turbine by this example (Fig. 4):

Wind turbine parameters as shown in Table 1:

**Table 1:** Turbine wind parameters.

Type of parameter	Value
Nominal power (*P*^*w*^)	1 kW
Speed cut in (*V*_*cin*_)	3.2 m/s
Speed cut out (*Vco*)	21 m/s
Rated wind speed (*Vrat*)	11.4 m/s
The price of capital (per kW)	2300 $
Price of installation (per kW)	1500 $
M & O price (per kW)	2 $/yr
Hub height	52 m
Total efficiency	26.5%
Lifetime	20 years

Related power is the power at the related wind speed with pitch angle beta = 0 and the turbine speed is the nominal generator speed.

To simplify the model and turbine functioning, we proposed the following hypotheses:

- The beta angle is always zero.
- The volumetric air mass and wind speed are not affected by altitude.
- P(V) is a linear function for Vc < V < Vr.

So, Pr = P(Vr).

We have (Fig. 5):

**Fig. 5:**
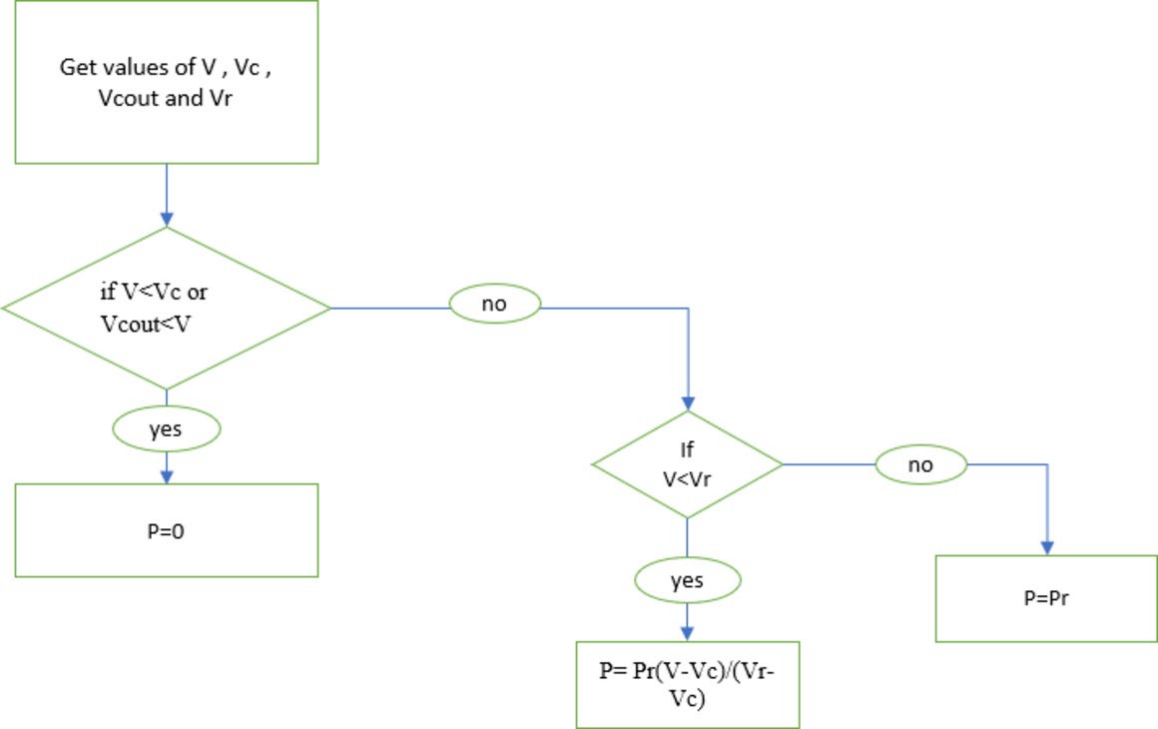
Flowchart of wind turbine.

### Solar panel

A solar panel’s output power is:

2 $$\text{P}= \text{A}.\text{G}\text{h}\left(\text{t}\right).\text{r}.\text{F}\text{l}\text{o}\text{s}\text{s}$$

We can also experiment with the following equation:

3 $$\text{P} =\text{P}\text{r}. \text{F}\text{l}\text{o}\text{s}\text{s}.\left(\text{G}\text{h}\right(\text{t})/\text{G}\text{s})$$

With Gs = 1000 W/m^2^.

The solar panel parameters are explained in the Table 2:

**Table 2:** Solar panel parameters.

Type of parameter	Value
Rated power (Ps)	1 kW
Cost capital (per kW)	1200 $
Price of installation (per kW)	1200 $
M & O price (per kW)	4 $/yr
Lifetime	20 years
Derating factor (floss)	88%

### Power converter

The utilization of converters of power between DC and AC^23^ is necessary in systems where both DC and AC components are present. In a microgrid that involves batteries and PV panels, DC output is generated while the given load is AC. Therefore, power converters are required to convert the DC output into AC output. The converter size is determined based on the peak load demand (PmðtÞ) of the system. The rating of the inverter (Pinv) can be found using the following Eq. (4):

4 $$\text{P}\text{i}\text{n}\text{v} = \text{P}\text{m} + \varDelta \text{P}$$

where Pm represents the peak load demand and ∆P represents the additional power required for losses in the conversion process. Therefore, to ensure that a system operates efficiently and effectively, it is crucial to select an appropriate converter size and calculate the correct inverter rating.

We can also model a power converter as below: Pinv = r.Pm.

Where is the efficiency.

Pm is the input power.

The converter parameters as shown in Table 3:

**Table 3:** Converter parameters.

Type of parameter	Value
Nominal power	150 kW
The efficiency	*r* = 90%
Cost of installation per kw	127 $/kW
M & O cost (per kW	1 $/Kw

### Battery

The power output of a battery depends on its state of charge (SOC), which is the amount of energy stored in the battery relative to its maximum capacity. The mathematical model for the output power of a battery in the function of SOC^24,25^ is as follows:

5 $$\text{P} = \text{V} * \text{I} * \text{f}\left(\text{S}\text{O}\text{C}\right)$$

The function f(SOC) varies depending on the type of battery chemistry and its discharge characteristics. For example, lead-acid batteries have a relatively flat discharge curve, meaning that their output power remains relatively constant over a wide range of SOC values. Lithium-ion batteries, on the other hand, have a more exponential discharge curve, meaning that their output power drops off more rapidly as SOC decreases.

To model f(SOC) for specific battery chemistry, manufacturers typically conduct extensive testing to measure the discharge characteristics of their batteries over a range of SOC values. They then use this data to develop an empirical equation that best fits the observed behavior.

The battery state of charge (SOC) is a measurement of the available capacity of a battery expressed as a percentage. The SOC can be calculated using various methods, including voltage, current, temperature, and impedance measurements. The most common method for calculating SOC is based on the battery’s open-circuit voltage (OCV). In this method, the SOC is estimated by comparing the OCV of the Battery

6 $$\text{S}\text{O}\text{C}=(\text{O}\text{C}\text{V}-\text{O}\text{C}\text{V}\_\text{m}\text{i}\text{n})/(\text{O}\text{C}\text{V}\_\text{m}\text{a}\text{x}-\text{O}\text{C}\text{V}\_\text{m}\text{i}\text{n}) * 100{\%}$$

where SOC is the battery state of charge in percentage, OCV is the open-circuit voltage of the battery, OCV_min is the minimum OCV value corresponding to 0% SOC, and OCV_max is the maximum OCV value.

The values of OCV_min and OCV_max depend on various factors such as battery chemistry, temperature, and age. Therefore, these values need to be determined experimentally or obtained from manufacturer specifications.

Parameters of the battery as shown in the Table 4.

**Table 4:** Battery parameters.

Type of parameter	Value
Nominal capacity (*C*)	360 Ah
Nominal voltage (*V*_*batt*_)	6 V
Max charging current (*Imax*)	18 A
Round trip efficiency (g*batt*)	92%
One unit capital price	167 $
Price of installation (per unit)	67 $
M & O price (per unit)	1.67 $/yr
SOCmin	20%
SOCmax	100%
Lifetime	5 years

### Utility grid

The utility grid equation represents the balance between the supply and demand of electrical energy in a power grid. It is used to ensure that the amount of electricity being produced by power plants matches the amount being consumed by consumers at any given moment. The conservation of energy principle, which holds that energy may only be transmitted or converted, is the foundation of the Eq. (7) below:

7 $$\text{S}\text{u}\text{p}\text{p}\text{l}\text{y} = \text{D}\text{e}\text{m}\text{a}\text{n}\text{d} + \text{L}\text{o}\text{s}\text{s}\text{e}\text{s}$$

Where Supply refers to the total amount of electricity being created by power plants, Demand refers to the total amount of electricity being consumed by consumers, and Losses refer to the amount of electricity.

For the equation to remain balanced, the supply must always equal the demand plus losses. If there is an imbalance between supply and demand, it can lead to blackouts or brownouts, which can have serious consequences for both consumers and power companies. To ensure that the utility grid equation remains balanced, power companies use a variety of tools and techniques, including load forecasting, demand response programs, and energy storage systems. Load forecasting involves predicting future electricity demand based on factors such as weather patterns and consumer behavior. Demand response programs encourage consumers to reduce their electricity usage during high-demand times, while energy storage systems allow excess electricity to be stored for later use. Overall, the utility grid equation is a critical component of modern power systems, ensuring that electricity is delivered reliably and efficiently to consumers around the world.

### Biomass gasifier

A biomass gasifier is a device that converts organic materials, such as wood chips, agricultural waste, or other biomass feedstock, into a combustible gas mixture known as producer gas^26^. This gas can be the fuel for various applications, including electricity generation, heating, and transportation.

The process of gasification involves heating the biomass feedstock in the presence of a limited amount of oxygen or air to produce a gas mixture consisting mainly of carbon monoxide, hydrogen, and methane^27^. The resulting gas can be used directly as a fuel or further processed to remove impurities.

Biomass gasification has several advantages over traditional fossil fuels. It is a sustainable and renewable energy source that can reduce dependence on foreign oil and greenhouse gas emissions. Additionally, it can provide economic benefits to rural communities by utilizing locally available biomass.

However, biomass gasification is associated with several challenges. The process requires careful control of operating conditions to ensure efficient conversion of the feedstock into gas. Furthermore, the composition of the producer gas can vary depending on the type and quality of the biomass.

Overall, biomass gasification has the potential to be crucial in meeting our needs for power while reducing our environmental impact.

Biomass gasification is a process that converts organic materials such as wood, agricultural waste, and municipal solid waste into a combustible gas mixture known as producer gas. This gas can be used to generate electricity through a gas engine or turbine^28^. The power output of a biomass gasifier can be found.

The formula of biomass gasifier is as follows:

8 $$\text{P}\text{o}\text{w}\text{e}\text{r}\:\text{O}\text{u}\text{t}\text{p}\text{u}\text{t} = \text{G}\text{a}\text{s}\:\text{F}\text{l}\text{o}\text{w}\:\text{R}\text{a}\text{t}\text{e} \times \text{H}\text{e}\text{a}\text{t}\text{i}\text{n}\text{g}\:\text{V}\text{a}\text{l}\text{u}\text{e} \times \text{E}\text{f}\text{f}\text{i}\text{c}\text{i}\text{e}\text{n}\text{c}\text{y}$$

The gas flow rate can be measured using a flow meter installed in the gas line. The heating value of the producer gas depends on its composition, which varies depending on the type of biomass feedstock and the operating conditions of the gasifier. Typically, producer gas has a heating value between 4 and 7 MJ/m^3^.

The efficiency of the system depends on various factors such as the design and operation of the gasifier, cleaning and conditioning equipment, and power generation equipment. A well-designed system has a 30%.

In summary, we can calculate the power output of a biomass gasifier by multiplying the gas flow rate by the heating value and efficiency. This equation can help you estimate the potential power output of a biomass gasification plant.

Biomass system parameters (Table 5):

**Table 5:** Biomass system parameters.

Type of parameter	Value
Nominal power	50 kW
Biomass calorific value (*CV*_*bm*_)	18 MJ/kg
Cost capital (per kW)	2300 $/kW
Price of installation (per kW)	1500 $/kW
M & O price (per kW)	2 $/yr
Lifetime	15,000 h

### The load

#### Dump load

The dump load equation^29^ is a mathematical formula used to calculate the amount of power that needs to be dissipated by a dump load to protect an electrical system from overvoltage or overcurrent conditions. Dump loads are typically used in energy-renewable systems, like wind turbines or solar panels, to avoid harming the system when excess energy is generated.

The dump load equation is:

9 $$\text{P} = \text{V}^{2}/\text{R}$$

This equation shows that the amount of power that needs to be dissipated by the dump load is proportional to the square of the voltage and inversely proportional to the resistance. Therefore, increasing the voltage or decreasing the resistance will result in a higher power dissipation requirement for the dump load.

In practical applications, it is important to choose a dump load with an appropriate resistance value^30^ based on the characteristics of the electrical system. If the resistance is too low, too much current will flow through the dump load and it may overheat or fail. If the resistance is too high, not enough power will be dissipated and the electrical system may still be at risk of damage.

#### Electric load

Electric load refers to the quantity of electricity that is being used by an electrical system at a given time. It is the total quantity of electricity used from the electrical grid by all connected devices and appliances. Understanding electric load is important for utilities to ensure that they are providing enough power to meet demand, and for consumer’s electric load can be categorized into two types: base load and peak load. Base load refers to the minimum amount of electricity required by a system over 24 h^31^. This includes power used by essential services such as hospitals, streetlights, and refrigeration systems. Peak load, on the other hand, refers to the maximum amount of electricity used in pic demand, for example on hot summer afternoons when air conditioning usage is at its highest. Electric load can also be managed through demand response programs. These programs incentivize consumers to reduce their energy usage during times of high demand in exchange for lower rates or other benefits. This helps utilities avoid blackouts or brownouts during peak periods.

The electric load equation is a mathematical formula that calculates the quantity of energy consumed by an electrical device or system. It is commonly used in electrical engineering and is essential in determining the power requirements for designing electrical systems^32^.

10 $$\text{P}=\sqrt{3}\:\text{U}\:\text{I}\text{ cos}\:\varphi$$

Where P is power in watts, V is voltage in volts, and I is current in amperes.

The electric load equation is based on the principle of Ohm’s law^33^, which states that the current through a conductor between two points is directly proportional to the voltage across the two points. The electric load equation helps to determine the power consumption of an electrical device or system and allows engineers to design and optimize electrical systems for maximum efficiency. In practical applications^34^, the electric load equation can be used to calculate the power requirements of various electrical devices such as motors, transformers, heaters, and lighting systems. Engineers can design efficient systems by understanding the power requirements of these devices.

**Fig. 6:**
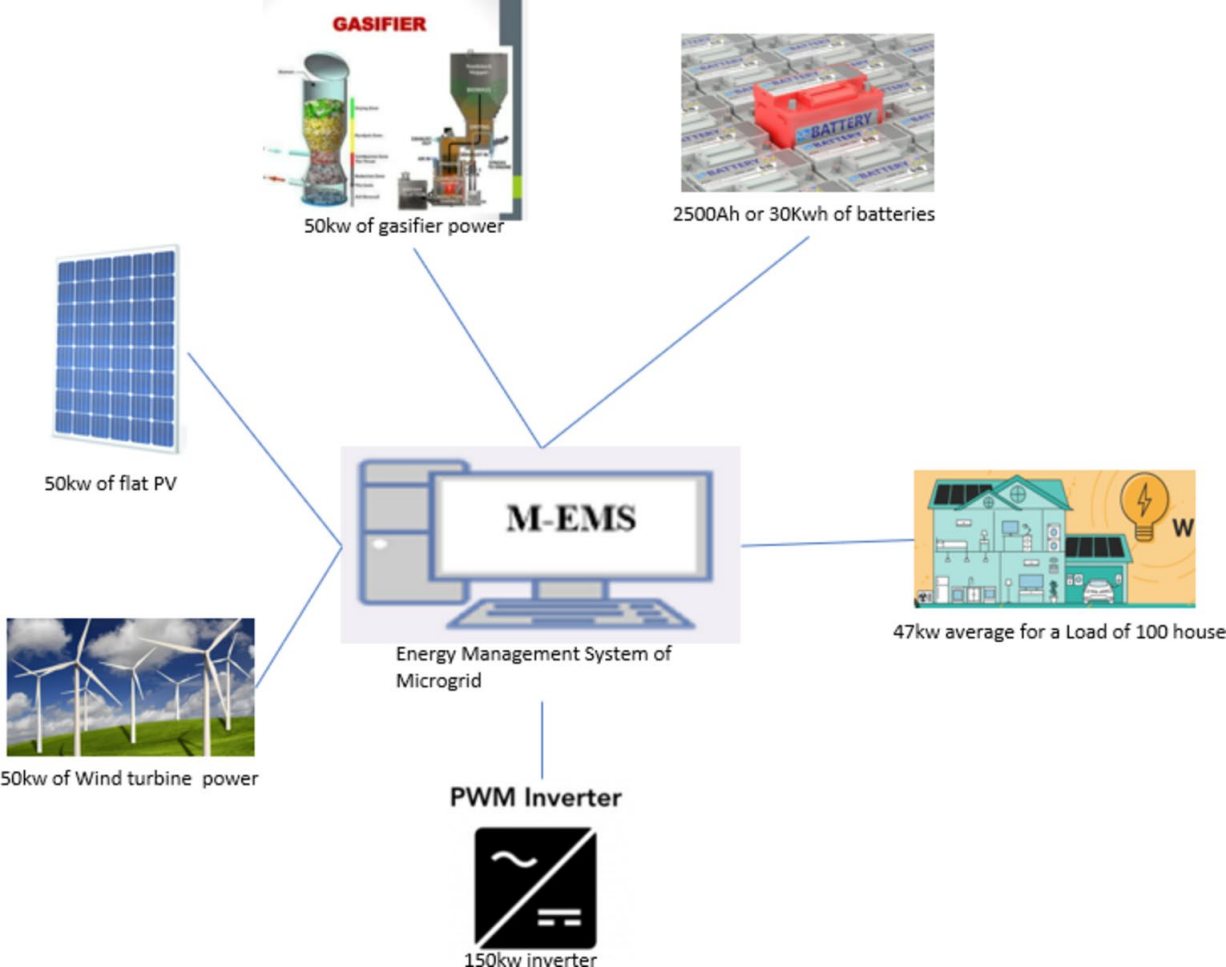
Microgrid estimated configuration.

In summary, the electric load equation is a basic concept in electrical engineering that helps to determine the power requirements of electrical devices and systems^35^. It plays a crucial role in designing efficient electrical systems that meet specific power demands (Fig. 6):

Estimated electricity demand for the community (Table 6).

**Table 6:** The estimated demand for electricity.

	Load type	Number	Power (W)	Summer h/day	(May–Sep) Watt-h/day	Winter h/day	(Oct–April) Watt-h/day
Domestic load							
1	CFL	4	22	10	880	7	620
2	CFL	1	12	8	96	11	132
3	Ceiling fan	1	121	20	2440	0	0
4	Kitchen fan	1	29	6	174	0	0
5	Cooler	2	121	10.5	2500	0	0
6	TV	1	99	9	890	6	591
7	PC	1	299	8	2392	9	2690
8	Exhaust fan	1	16	5	80	3	48
9	Table fan	1	16	8	124	0	0
10	Room heater	1	92	0	0	12	1104
11	Room heater	1	148	0	0	7	1036
12	Electric blanket	1	125	0	0	3	375
13	Vacuum cleaner	1	215	2	430	1	215
14	Bulb	1	102	1	102	2	204
	Total (one house)				10,143		6976
	Total all houses	100					
Overall demand (kWh/day)					1014		

### Microgrid controller

**Fig. 7:**
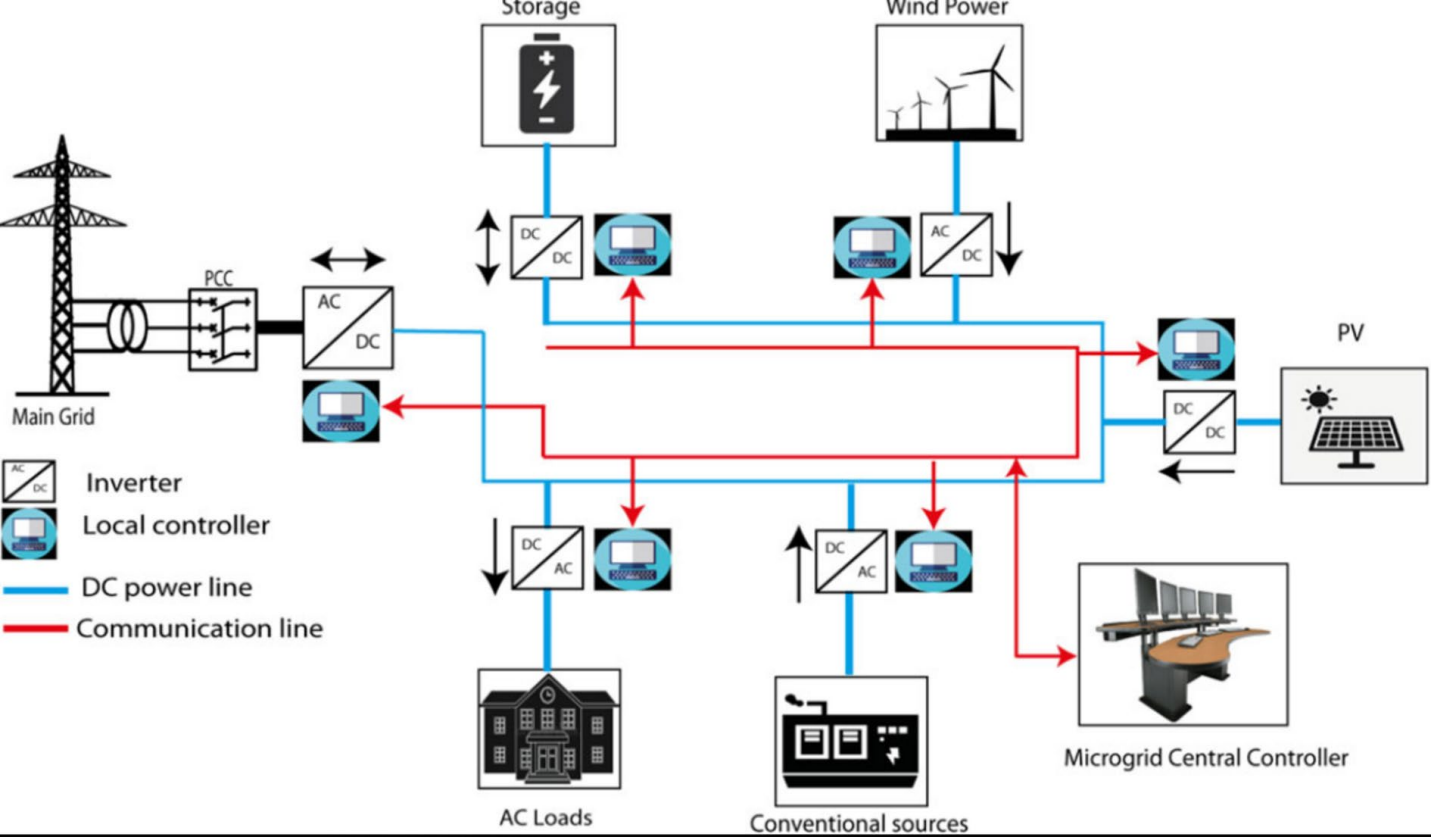
Microgrid controller.

A microgrid controller^36^ is responsible for managing and optimizing a microgrid’s operation, a small-scale power grid that can in conjunction or independently operate with the electrical main grid. The controller needs to manage the generation, storage, and consumption of energy optimally while considering various constraints and objectives. Here is a simplified mathematical model of a microgrid controller (Fig. 7).

Minimize the operational cost of the microgrid over a given time horizon while maintaining grid stability, meeting load demands, and satisfying generation and storage constraints.

This optimization problem can be solved using various techniques, such as linear programming or mixed-integer linear programming, depending on the complexity of the cost functions and additional constraints. The solution provides optimal power generation, storage, and consumption schedules for the microgrid controller to follow.

## Microgrid sizing

### Load size

A microgrid’s dimensions^37^ are determined by two fundamental factors: the size of the load and the natural resources that are available where the microgrid is located.

We will work on an average of 100 houses.

The total load is between 850 and 1200 kWh/day and the average is 1100 kWh/day.

**Fig. 8:**
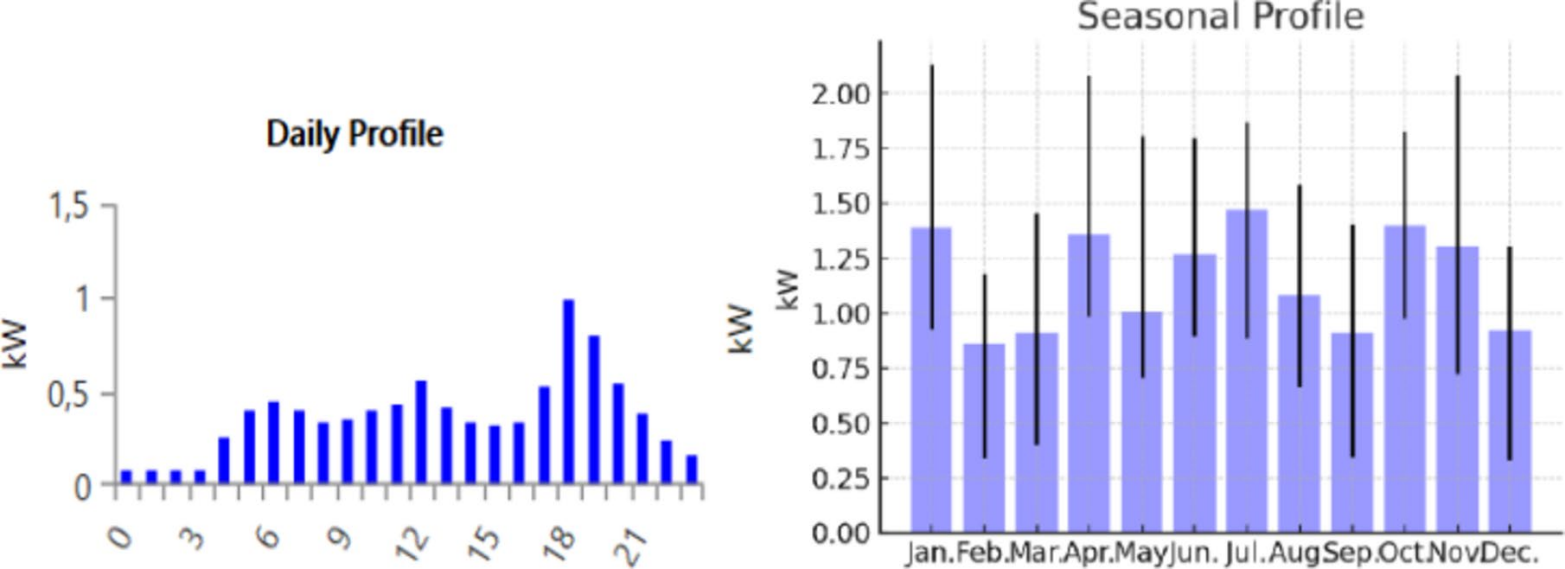
The power of one house.

**Fig. 9:**
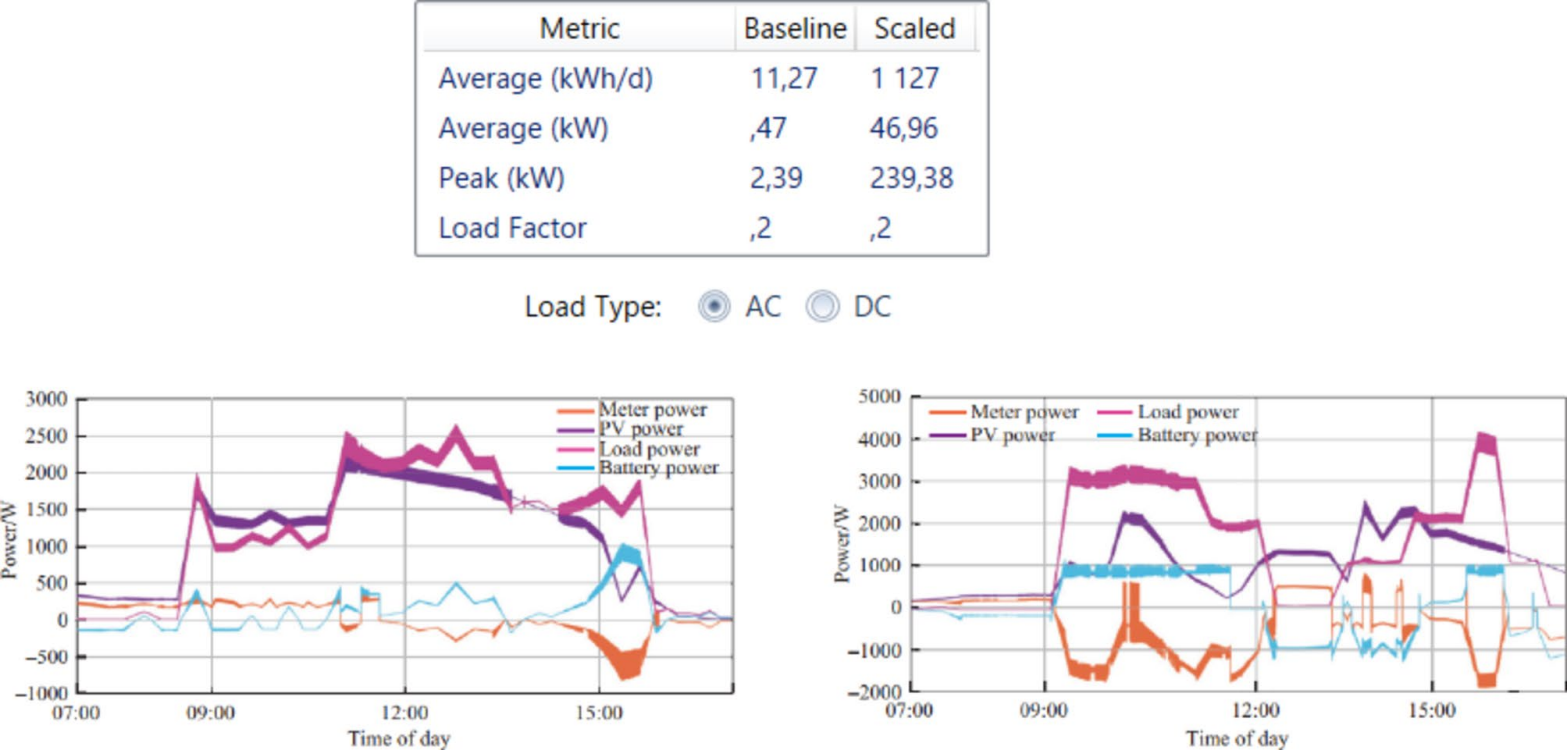
Domestic electricity power variation.

The graph represents the power of 1 house (Figs. 8 and 9).

**Fig. 10:**
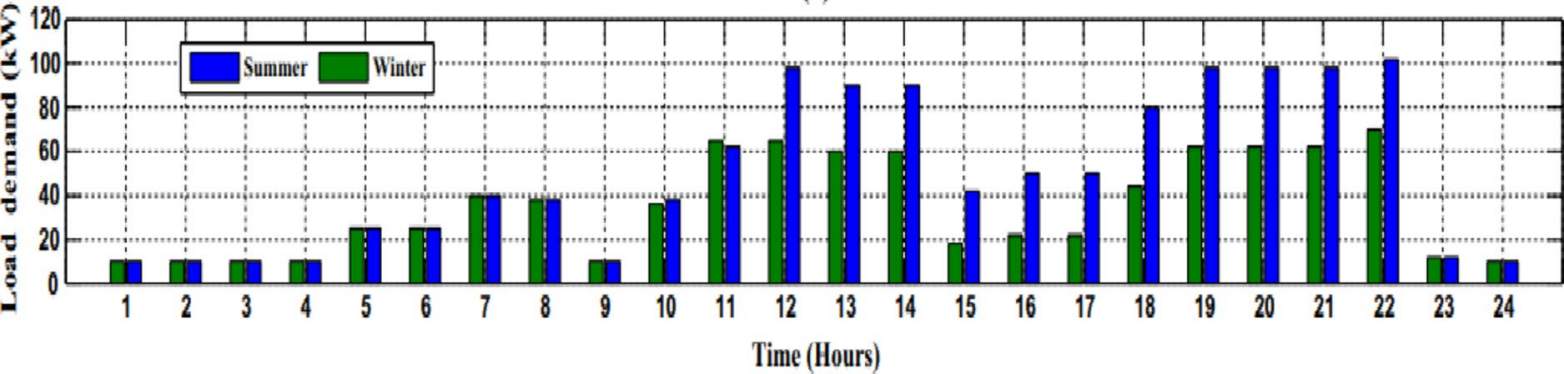
The load demand of 100 houses.

So, the peak load power is 240 kW and the average load power is 47 kW the hourly minimum is 17 kW and the maximum is 100 kW (Fig. 10).

The dynamic load Figs. 8, 9 and 10 above size is central to the successful sizing of a microgrid because it dictates the capacity of the system to meet variable demand. Microgrids will operate in isolation or in synchronism with the grid, and an accurate dynamic load profile measure ensures that the system will be able to adjust to real energy consumption variations. This figure takes into account not only the peak load, but also the varying demands resulting from weather, day of week, and operating status. Microgrid sizing based on these dynamic load profiles will ensure that generation and storage units are not over- or under-sized, hence enhancing better efficiency, cost effectiveness, and system reliability. It enables the microgrid to adapt to rapid rises or drops in demand without compromising on performance or safety.

### The natural resources in Fez

#### Wind resources

**Fig. 11:**
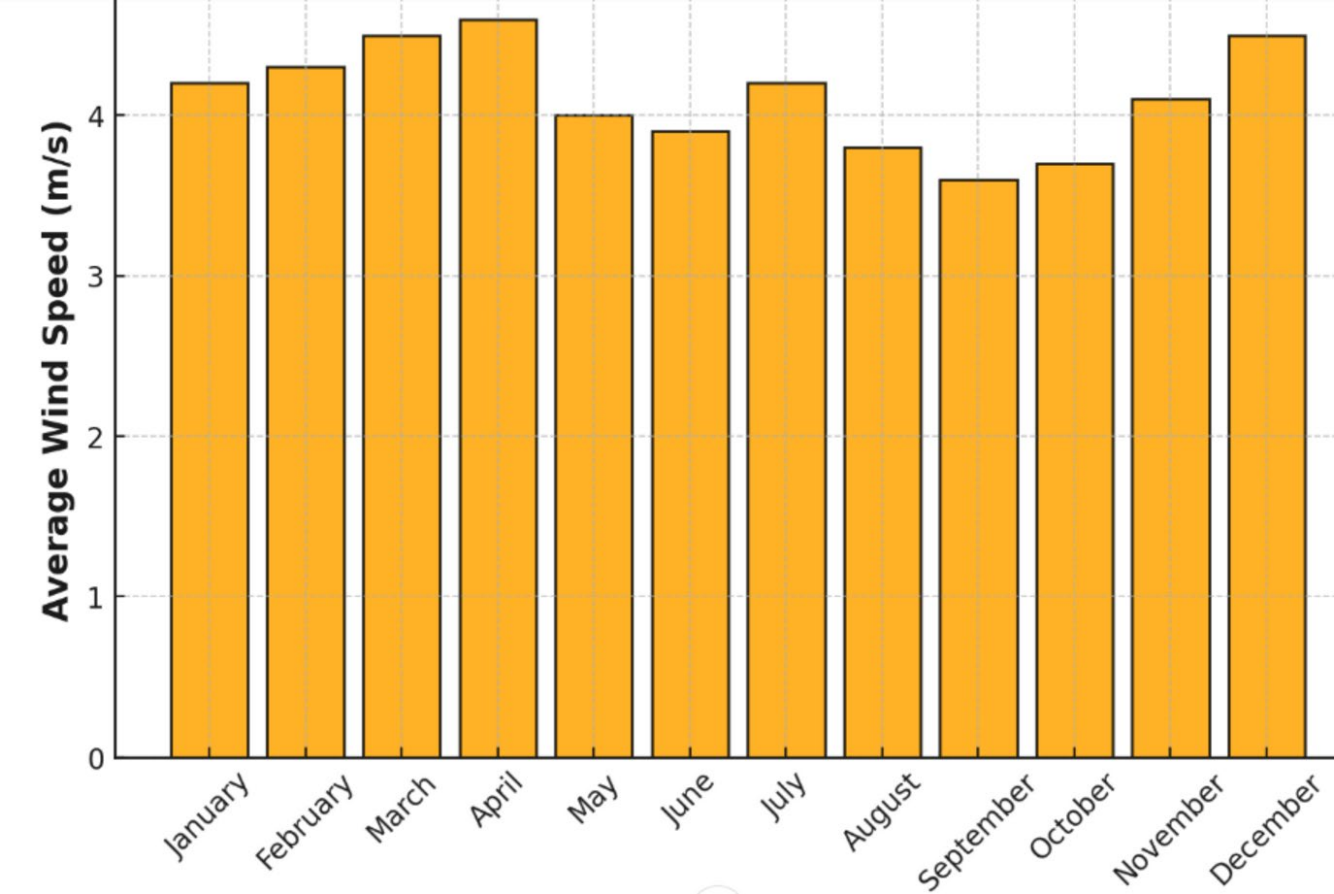
Wind speed monthly basis.

We noticed that the wind speed for the turbine is close to the 3 m/s vcin speed even though the turbine needs to be moving at a speed of 11 m/s to provide its nominal power. Hence, atmospheric conditions do not promote the wind (Fig. 11).

If one applies the relationship between the output power and the wind speed and uses the average speed of 4 m/s available to them, they will obtain p = (4 − 3)/(11 − 3) Pn. P = (1/8) Pn.

According to the following relationship^38^, the wind speed changes as a function of height. V = Vr(H/Hr)^(1/7). It implies that, according to the following relationship, the wind power will vary with altitude: P = Pr(H/Hr)(3/7), so for a height of 5 h, one will either have *P* = 5(3/7) Pr or *P* = 2Pr.

#### Solar resources

**Fig. 12:**
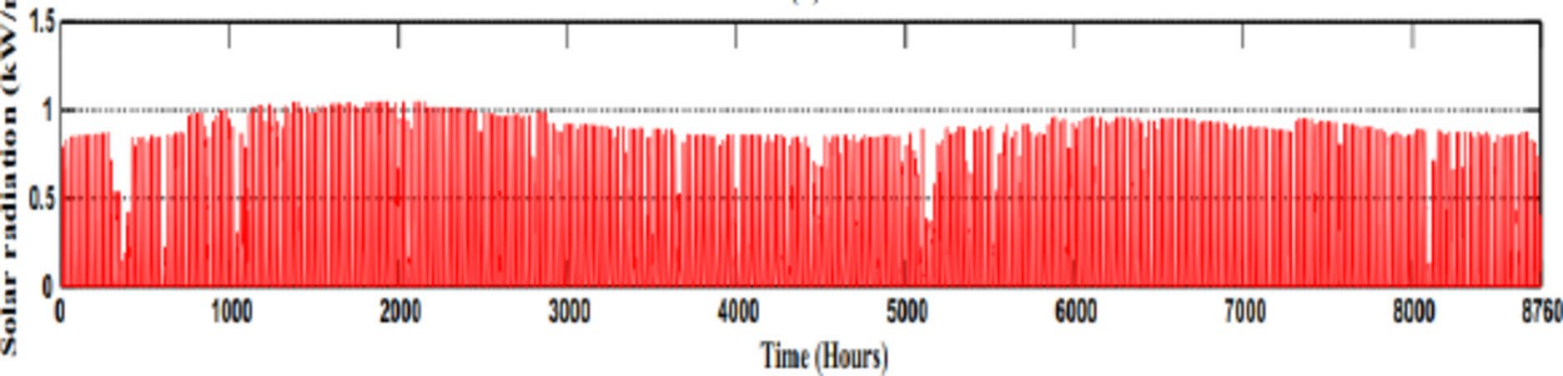
Solar power in a year.

Taking into account a day of 12 h of sunlight, we will see that the average power in W/m^2^ is 417 W/m^2^. The maximum power of solar radiation is about 1 kW/m^2^ as shown in the graph below (Fig. 12).

**Fig. 13:**
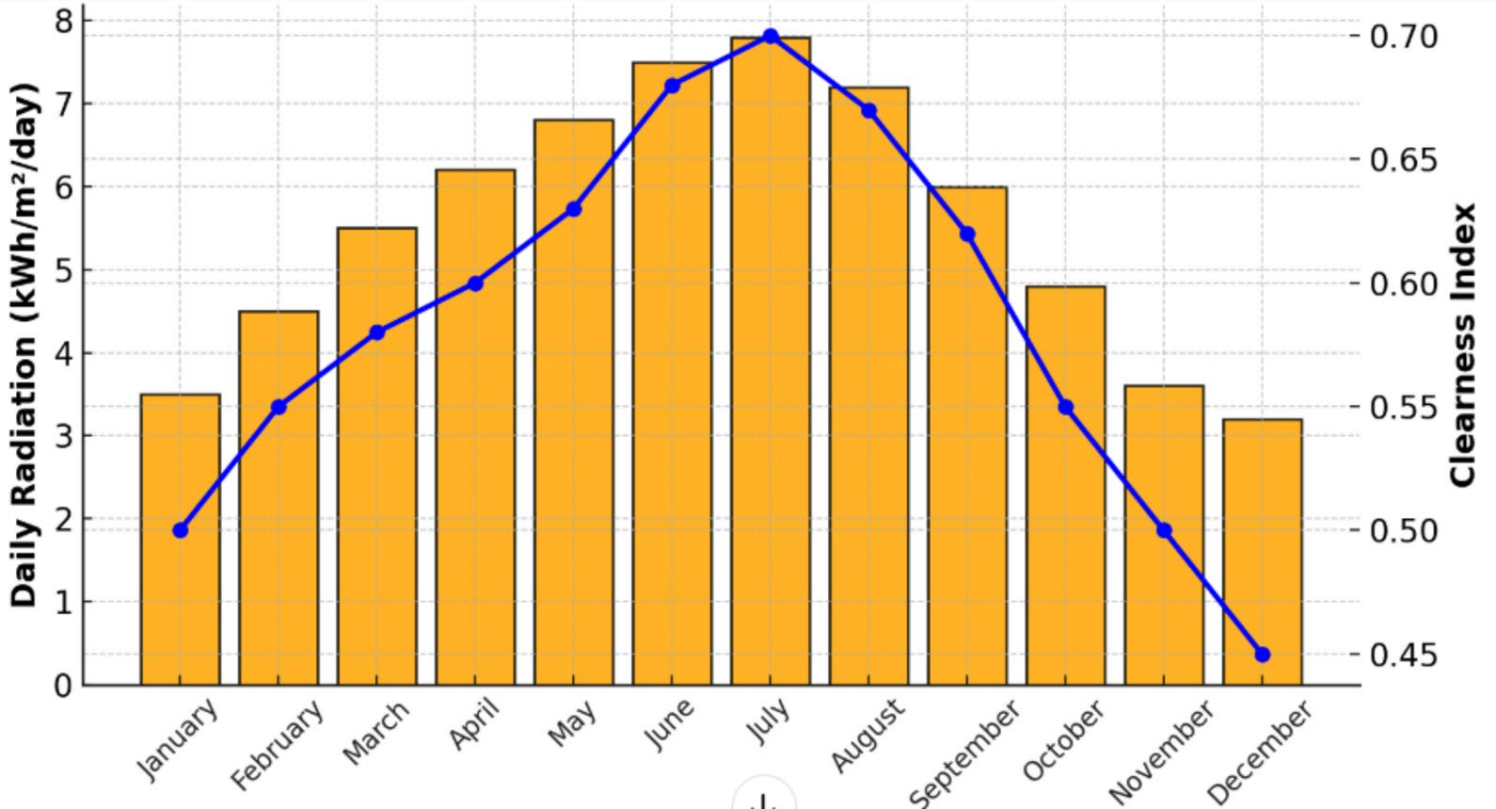
Solar energy monthly basis.

The Clearness Index^39^ is an important parameter in many fields, including solar energy, meteorology, and climate science. It is used to estimate the quantity of solar energy that can be harvested by solar panels at a particular location, as well as to study the effects of clouds and atmospheric aerosols on climate (Fig. 13).

When the power output to radiation ratio for the solar panel is applied while taking into account the average radiation at fez, the result is p = Pn (417/1000). So, *p* = 0.42 Pn.

Figures 11 and 12, and 13 as shown above indicate the microgrid inputs of natural resources based on wind speed and solar irradiance. Such inputs are essential for guaranteeing the optimal operation and design of a microgrid by providing necessary data for accurate sizing and choice of the components. By analyzing wind speed and solar irradiance trends, we can determine the cheapest and most effective energy generation options to ensure the microgrid can meet the energy demand while maximizing the utilization of renewable resources. Sizing the equipment, such as wind turbines, solar panels, and energy storage systems, based on these natural resource inputs creates a more sustainable, reliable, and economically viable microgrid^40–45^.

### Results of sizing

We will use the average value of 47 kW to dimension because we use the utility grid, batteries, and biomass.

The maximum number of solar panels is: Np = 47/0.42; Np = 112 taking into account the inverter efficiency we will have Np = 112/0.9 so Np = 125. The maximum number of wind turbines is: Ne = 47/ (1/8) so Ne = 376 we will take into consideration an altitude of 5 h of 50 m so we will have Ne = 188 We will use wind turbines with a nominal power of 10 kW so we will obtain Ne = 19. For the batteries: The solar panels are not functional at night, necessitating the use of batteries. The average amount of energy required to deliver our load for a 12-h night is 550 kWh, so we will need 7 360-amp-hour batteries, to obtain 550,000/220 = 2500 Ah taking into account the SOC and efficiency we will have 20% SOCmin. *N* = 7/(0.8^2^), *N* = 14. For the biomass, we will have 1 biomass of 50 kW.

Same thing for the inverter 115 kW because our load average puissance is 47 kW. So, Np < 125; Ne < 19; Nb < 14 with Panel has 1 kW; wind turbine 10 kW and batteries 360 Ah.

## Formulation problem

The creation of a dependable and economical hybrid energy system^46^ is the first objective of this project. The rating and size of solar PV panels, wind turbines, battery banks, and biomass gasifiers are the main determining criteria. The system’s operational plan is covered in this section.

This study’s primary goal is to reduce the total NPC of the proposed hybrid system while preserving the best energy efficiency.

The quantity^47^ of PV panels, batteries, biomass gasifiers, and wind turbines has been decided. The annualized system cost (ASC) idea is employed for the economic analysis. While meeting all other constraints and criteria, it is seen that the result with the lowest ASC is the best one. The considered objective function is the overall system cost, which is made up of (i) total capital costs, (ii) replacement costs, (iii) the expense of operating and maintaining the components.

$$\text{Minimize: } \text{A}\text{S}\text{C}= \text{F}(\text{N}\text{s}\text{o}\text{l}\text{C}\text{s}\text{o}\text{l}+\text{N}\text{w}\text{t}\text{C}\text{w}\text{i}\text{n}\text{d}+\text{N}\text{b}\text{a}\text{t}\text{t}\text{C}\text{b}\text{a}\text{t}\text{t}+\text{P}\text{i}\text{n}\text{v}\text{C}\text{i}\text{n}\text{v}+\text{P}\text{b}\text{m}\text{g}\text{C}\text{b}\text{m}\text{g})$$

where Csol, Cwind, Cbatt, and Cinv are the prices for batteries, solar PV panels, wind turbines and inverters, respectively, per kW.

**Fig. 14:**
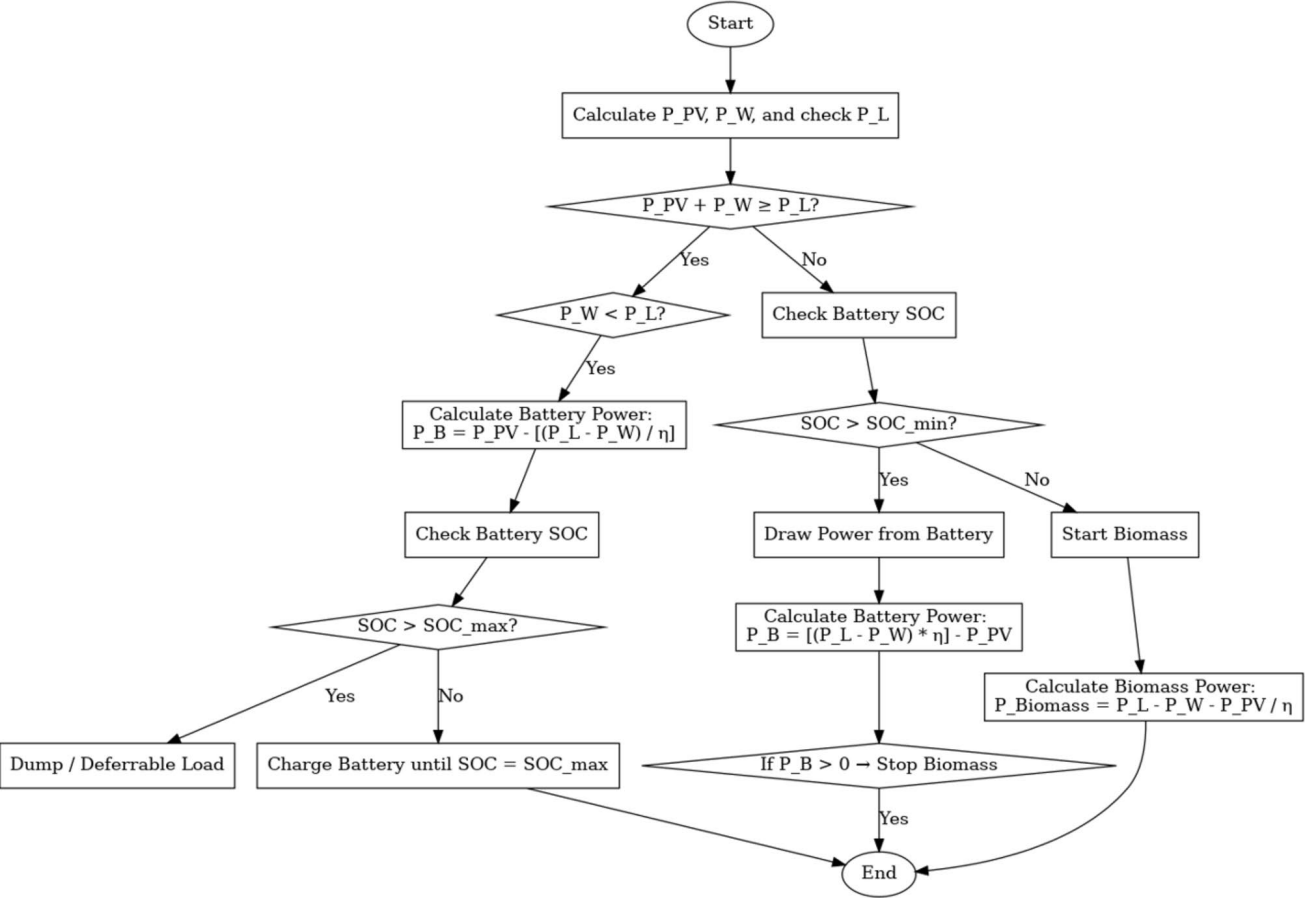
Flowchart of microgrid functioning.

The outline operating plan of the planned energy hybrid system can be announced in (Fig. 14).

To model the price per kilowatt-hour (kWh) of a microgrid, we need to consider various parameters like the initial investment price, operational and maintenance costs^48^, fuel costs (if applicable), and any subsidies or incentives available. Additionally, the pricing structure may vary depending on the specific location.

It is important to note that the price per kWh may also vary based on different tariff structures, such as time-of-use pricing or demand-based pricing, which can further influence the overall cost for consumers.

So, our mathematic model to optimize is:

$$(\text{N}\text{w}\text{i}\text{n}\text{d}.\text{C}\text{w}\text{i}\text{n}\text{d} + \text{N}\text{p}\text{a}\text{n}\text{e}\text{l}.\text{C}\text{p}\text{a}\text{n}\text{e}\text{l} + \text{N}\text{b}\text{a}\text{t}\text{t}.\text{C}\text{b}\text{a}\text{t}\text{t} + \text{C}\text{o}\text{n}\text{v}\text{e}\text{r}\text{t} +\text{C}\text{b}\text{i}\text{o}\text{m}\text{a}\text{s}\text{s})/({<} \text{P}\text{m}\text{o}\text{y}{>})$$

With <Pmoy> = 47 kw. 365.24 0.20 = 8,2 Gwh.

<Pmoy> = Nwind <Pwind> + Npanel <Ppanel>.

Cpanel = 1200 + 1200 + 4*20 = 2480usd/kw.

Cwind = 2300 + 1500 + 20*4 = 3880usd/kw.

Cbatt = 234*4 + 1.67 + 20 = 970 usd/unit.

Cconvert = 150*20 + 127*150 = 22000usd.

Cbiomass = 3880*50 = 194000usd.

And Nwind < 19; Npannel < 125; Nb < 14.

**Fig. 15:**
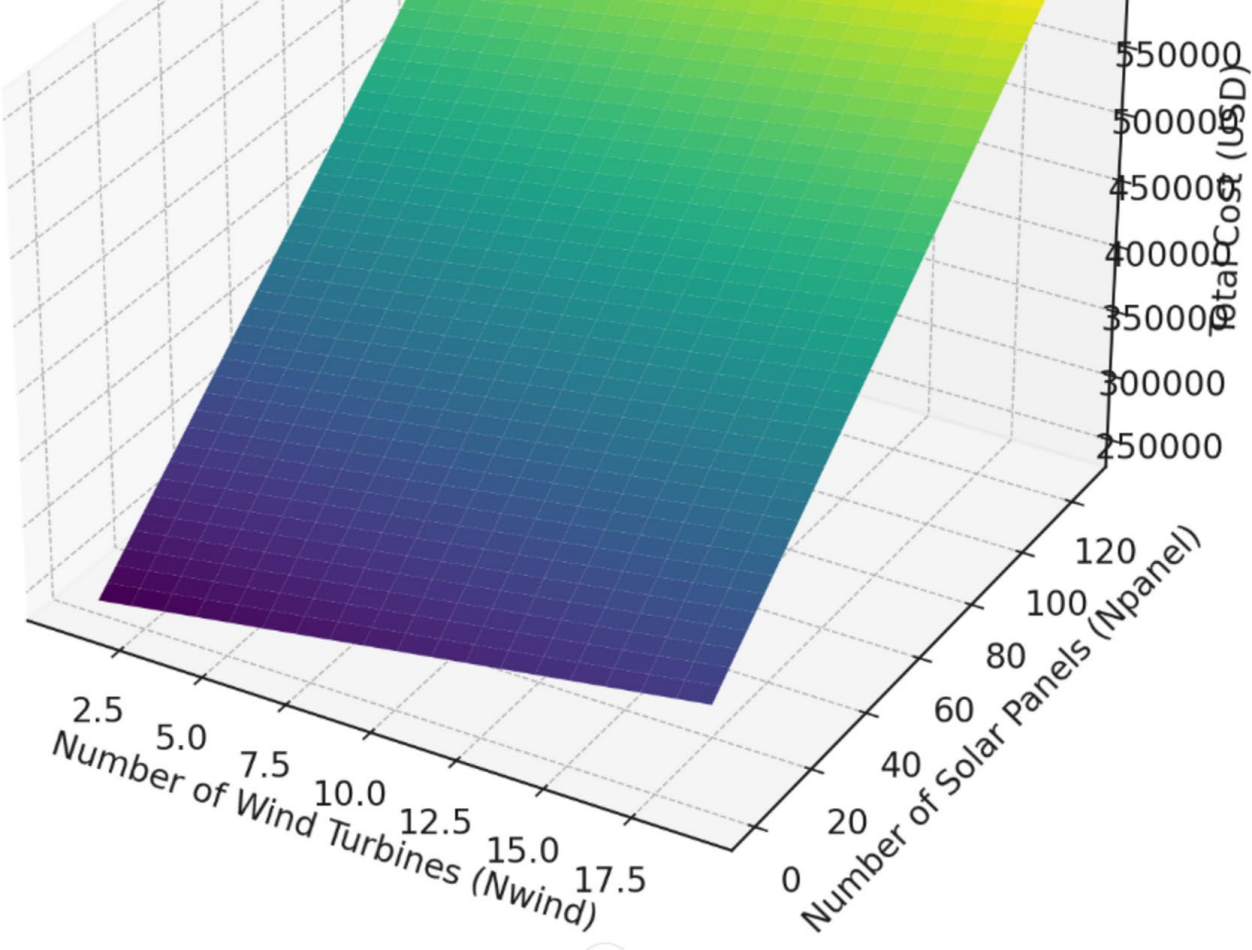
The microgrid cost visualization.

To explain better our mathematic model here’s a 3D surface plot visualizing the total cost of the energy system based on the number of wind turbines (Nwind) and solar panels (Npanel) as shown in the Fig. 15. The cost increases as the number of turbines and panels increases, with the z-axis representing the total system cost in USD.

- The x-axis represents the number of wind turbines.
- The y-axis represents the number of solar panels.
- The z-axis shows the corresponding total cost for each combination of wind turbines and solar panels.

We can observe how the cost rises with increasing numbers of turbines and panels, which aligns with the model provided.

So, our objective is to resolve the quadratic problem using different AI approaches and find the optimum Npanels and Nwind (Nwind, Npanel) optimum point that will give us minimum costs while respecting the microgrid average need on power, which is 47 kW, and also taking into consideration different costs of components. Their life, their maintenance costs, and also the available geographic resources.

## Resolution using AI methods

### Genetic algorithm

A search and optimization method called a genetic algorithm draws inspiration^49^ from natural selection and evolution. It is used to simulate the survival of the fittest within a population of potential solutions (individuals) to identify solutions to optimization problems.

A population of randomly created people serves as the initial population for the genetic algorithm. Every person is a potential response to the optimization issue. The algorithm then assesses each person’s fitness using the objective function. The fitness function measures how well each person performs concerning the optimization aim (in this example, minimizing the objective function).

To produce a new population through processes like selection, crossover^50^ (recombination), and mutation, the algorithm then chooses individuals based on their fitness. Higher-fitness individuals are more likely to be picked as parents for the following generation due to the selection process’ preference for them. To create offspring, parents are combined in a process known as crossover, whereas a mutation alters a gene at random.

The same assessment and selection procedures are used for the new population, also known as the offspring generation. For a predetermined number of generations or until a termination condition is satisfied, this cycle of assessment, selection, and reproduction continues.

In the case of multi-objective issues, the genetic algorithm seeks to identify a group of individuals that best represents the trade-off between objectives or, in the case of single-objective problems, the best solution.

**Fig. 16:**
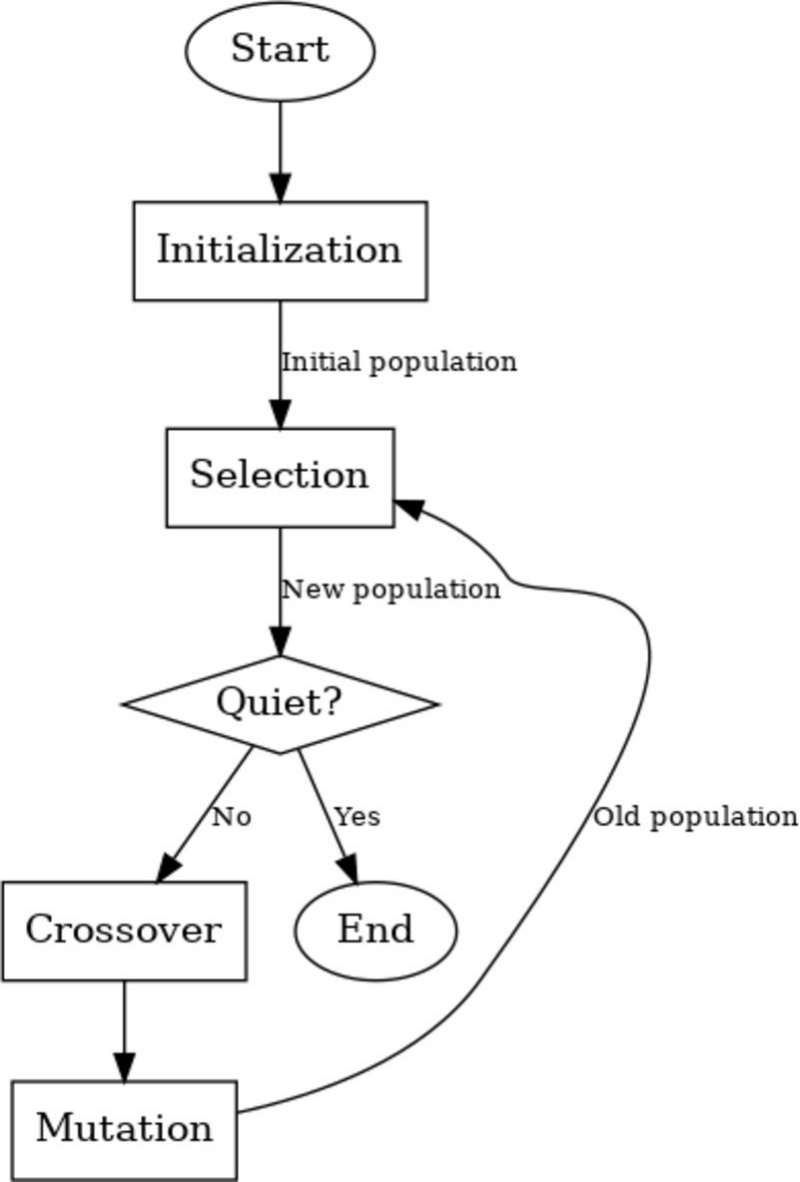
Genetic algorithm flowchart.

Below is a flowchart illustrating the steps of the genetic algorithm (Fig. 16).

1. Initialization is the process of randomly creating a population of potential solutions (individuals). Each person is represented as a collection of genes, which serve as the problem’s decision factors.
2. Evaluation: Assess each population member’s level of fitness. Each solution’s performance concerning the objective function is quantified by the fitness function. It directs the genetic algorithm’s selection procedure.
3. Selection: To produce the next generation, choose individuals from the current population. The selection process is biased in favor of people who are more physically fit since they are more likely to be chosen as parents.
4. Reproduction (Crossover): Crossover, sometimes called recombination, is the process of producing offspring from chosen parent individuals. Crossover produces new people by fusing the DNA of two parents, adding diversity to the population.
5. Mutation: To maintain genetic diversity, introduce random changes (mutations) into a few of the offspring’s genes. Exploration of new areas of the search space is made possible through mutation.
6. Replacement: Introduce the new generation of children to replace the previous one. The population for the following iteration is formed by this.
7. Termination: Repeat steps 2 through 6 until a termination condition is satisfied or for a predetermined number of generations. An acceptable answer or exceeding the maximum number of iterations could be the termination condition.
8. Solution: As the answer to the optimization issue, return the best individual (or individuals) discovered in the last generation.

The genetic algorithm iteratively evolves the population over several generations, with each generation potentially producing better solutions. The algorithm aims to converge to the best possible solution(s) over time.

The solution using a genetic algorithm is:

Wind Turbines number (Nwind): 5.

Number of Solar Panels (Npanel): 50.

Number of Batteries (Nbatt): 3.

Cost of the system: 0.035 USD/kWh.

### Ant colony optimization

**Fig. 17:**
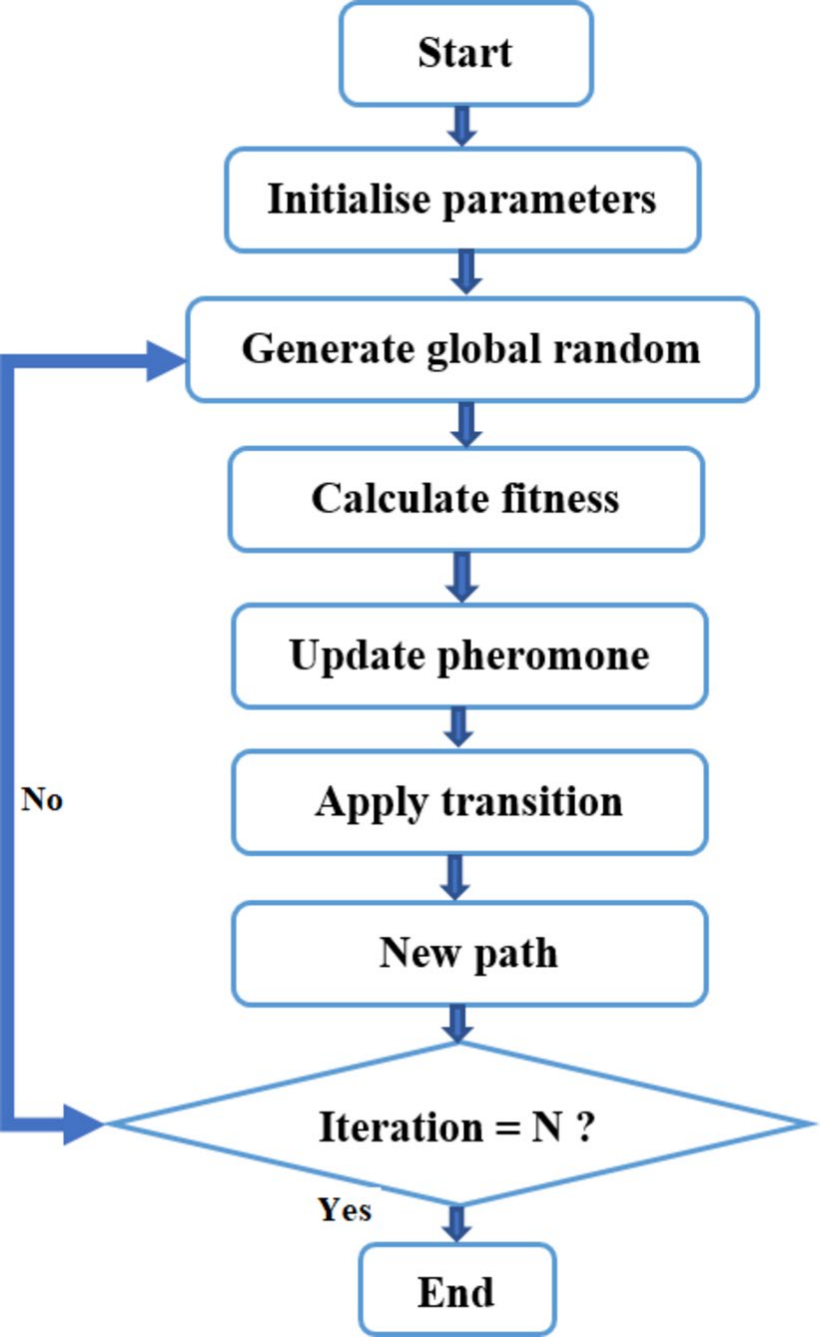
Ant colony flowchart.

ACO is a metaheuristic optimization^51^ algorithm that draws inspiration from and the foraging behavior. Since its invention by Marco Dorigo at the beginning of the 1990s, it has been extensively employed to resolve combinatorial optimization issues. The technique works particularly well for situations involving determining the optimum path or route through a network or graph. It works following those steps (Fig. 17):

1. Ants and Environment: The method is based on a figurative representation of how an ant colony interacts with its surroundings. The issue is depicted as a graph, where nodes stand in for cities or other places, and edges for the routes that connect them.
2. Pheromone Trails: Each edge in the graph has an associated pheromone value^52^. Ants deposit pheromone on the edges they traverse, and the intensity of pheromone on an edge is proportional to the quality of the solution found. Pheromone^53^ trails evaporate over time, representing the decay of the information.
3. Solution Construction: Ants construct solutions by probabilistically choosing paths based on the pheromone levels and a heuristic value (usually based on the distance or cost between nodes). The probability of selecting an edge is influenced by both the pheromone level and the attractiveness (heuristic information) of the edge.
4. Solution Improvement: As ants construct solutions, they update the pheromone levels on the edges based on the quality of the solutions found. Better solutions receive more pheromone deposits, which, in turn, increases the probability of selecting those edges in future iterations.
5. Exploitation vs. Exploration: ACO balances the trade-off between exploitation (favoring high-quality solutions) and exploration (searching for new solutions) through the use of pheromones and heuristic information. The algorithm encourages the exploitation of paths with higher pheromone levels while also allowing some exploration of lesser-known paths.
6. Iterative Process: The algorithm repeats the solution construction, s evaluation, and pheromone update process for a specified number of iterations or until a termination condition is met.
7. Global Best Solution: During the iterations, the best solution found so far is recorded, and it represents the best solution found by the colony of ants.

Its application to optimize our function is:

- defining the problem’s parameters, including its costs and constraints.
- Initialize the decision-making phenomenological matrix (Nwind, Npanel, and Nbatt) with positive a priori randomized values.
- Make the best solution’s cost and value (best_cost and best_solution, respectively) be defined as zero and infinite.
- Repeat for a set number of repetitions.
- Create a solution for each ant in the colony using the probability based on the heuristic information and pheromones.
- Updating the best solution and its price while determining if the proposed solution is superior to the best option currently available.
- Updating the phenomenometric matrix by the colony’s best solution.

After the iterations, we obtain the best solution.

The most optimal solution:

Number of Wind Turbines (Nwind): 5.

The Solar Panels number (Npanel): 50.

The Batteries number (Nbatt): 3.

Cost of the system: 0.035 USD/kWh.

### ABC algorithm

A swarm intelligence optimization method that draws inspiration from honey bees’ feeding habits is the Artificial Bee Colony (ABC) algorithm^53,54^. Employed bees, observer bees, and scout bees are the three different kinds of artificial bees used in this algorithm to represent the three stages of the optimization process. To discover and utilize the search space, each bee carries out a distinct mission.

**Fig. 18:**
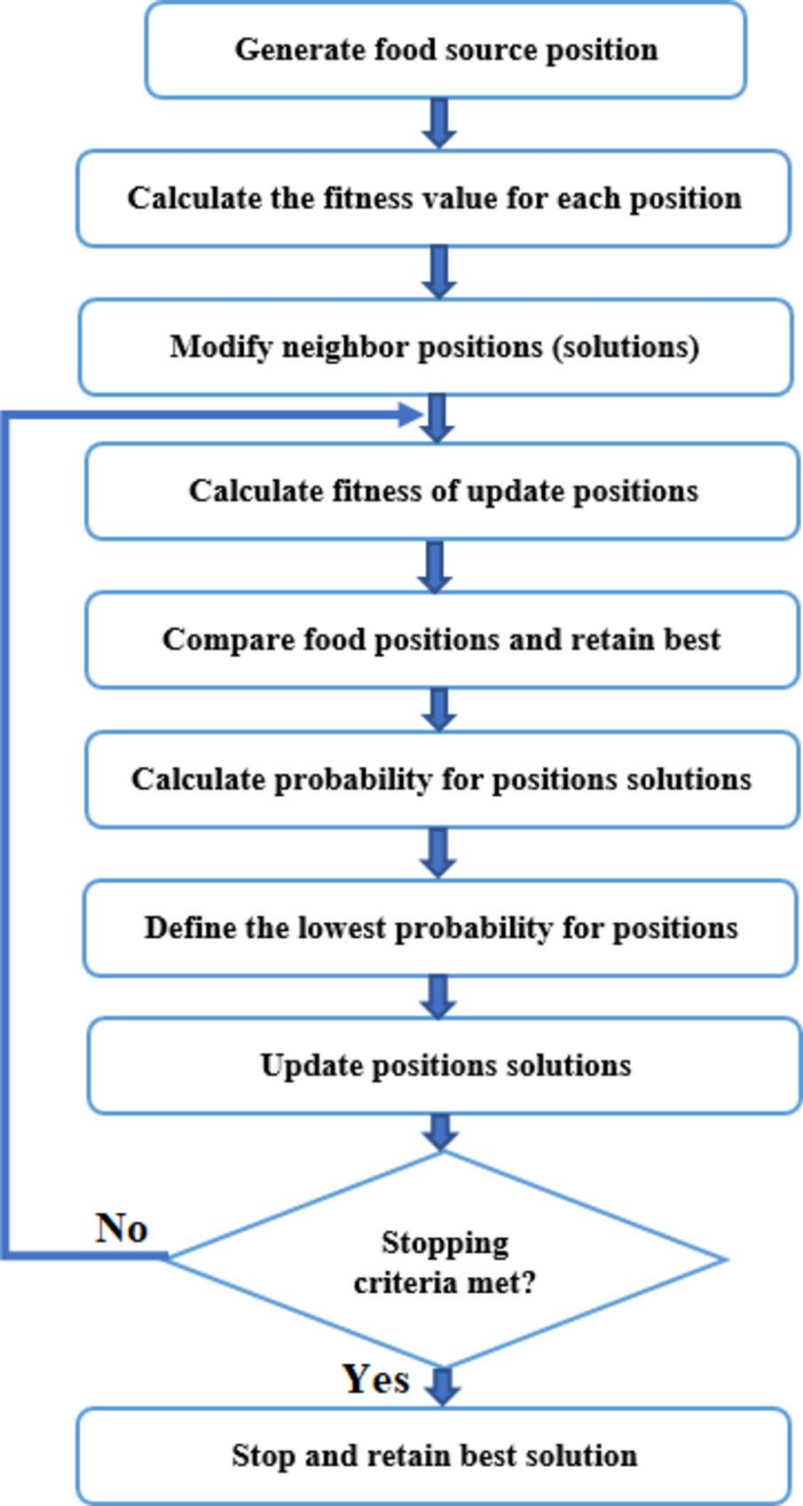
ABC flowchart.

The steps of the ABC algorithm are as follows (Fig. 18):

1. Initialization.

In the initialization phase, we begin by producing a population of synthetic bees, or “scout bees.” Every scout bee is a potential answer to our optimization issue. The scout bees are given these answers at random. These solutions’ quality is assessed using an objective function.

To solve our optimization problem, we start by building a colony of scout bees, each of which possesses a unique solution. We determine the quality of these first solutions by feeding them through our objective function after they are generated at random.

2. Employed bees’ phase:

The hired bees are now actively looking for better answers. Each employed bee chooses a neighbor bee to share information with and then creates a new solution based on this information. The effectiveness of these novel solutions is then assessed.

The hired bees are now working to make their solutions better. They select a nearby bee with whom they may share information, which enables them to revise their existing solution. We evaluate the novel solutions produced by this collaboration to identify their potential.

3. Onlooker bees’ phase:

Based on the caliber of the solutions found by the hired bees, onlooker bees decide which solutions to investigate. The fitness value of a solution determines the likelihood of choosing it. They come up with fresh ideas and assess their merit.

The bees in the audience are interested in the solutions developed by the working bees. Higher-quality solutions are preferred by them, and they are more likely to be chosen. They then come up with alternate options and evaluate their quality.

4. Scout bees’ phase:

Scout bees find “abandoned” solutions during this phase if they haven’t improved after a predetermined number of iterations. To investigate other areas of the search space, they swap out these abandoned answers for fresh, arbitrary ones.

The scout bees are essential for preserving population diversity. They keep an eye out for solutions that appear to be static or ineffective and swap them out for new, randomly generated ones. This supports a search for space exploration.

Employed bees, observer bees, and scout bees collaborate to enhance the solutions and converge towards an optimal or nearly optimal solution for the given optimization issue as the ABC algorithm cycles through these four steps repeatedly.

The outcome, the finest possible option globally, and its price.

The ABC algorithm’s most ideal answer is:

7 wind turbines total (Nwind).

38 solar panels total (Npanel).

3 batteries (Nbatt), total.

The system costs 0.037 USD/kWh.

## Discussion of solutions

### Most expensive solution using AI

To discuss our solutions, let’s use a genetic algorithm to find the most expensive solution (worst solution).

Using a genetic algorithm, we can find that the most expensive solution is

Most expensive solution:

Number of Wind Turbines (Nwind): 19.

Number of Solar Panels (Npanel): 125.

Number of Batteries (Nbatt): 14.

Cost of the system: 0.055 USD/kWh.

The ant’s colony optimization algorithm confirms the results above.

### Price of electricity in Fez

In Morocco, Fes, the average price of 1kwh is: C = 0.115 USD /kWh.

### Homer results

Using Homer, we obtain the results as follows:

The price using Homer is 0.264 USD/kWh.

Homer takes into account the peak value of the load, which is 210 kW so for the average value of the load of 47 kW we will obtain.

C = 0.264 * 47/210 so C = 0.06usd/kwh.

### Comparison of results

As we can see on the Table 7.

**Table 7:** Different results using different methods.

Method	Cost (USD/kWh)
Genetic Algorithm (Best)	0.035
ABC Algorithm	0.037
Genetic Algorithm (Worst)	0.055
Homer (Average Load)	0.06
Average Price of Electricity (Fez)	0.115

This table compares the system costs using different methods with the local electricity price in Fez, Morocco.

The cost of electricity can be reduced from 0.115 USD/kWH to [0.037 0.055] USD/kWH by using renewable energy sources alone.

**Fig. 19:**
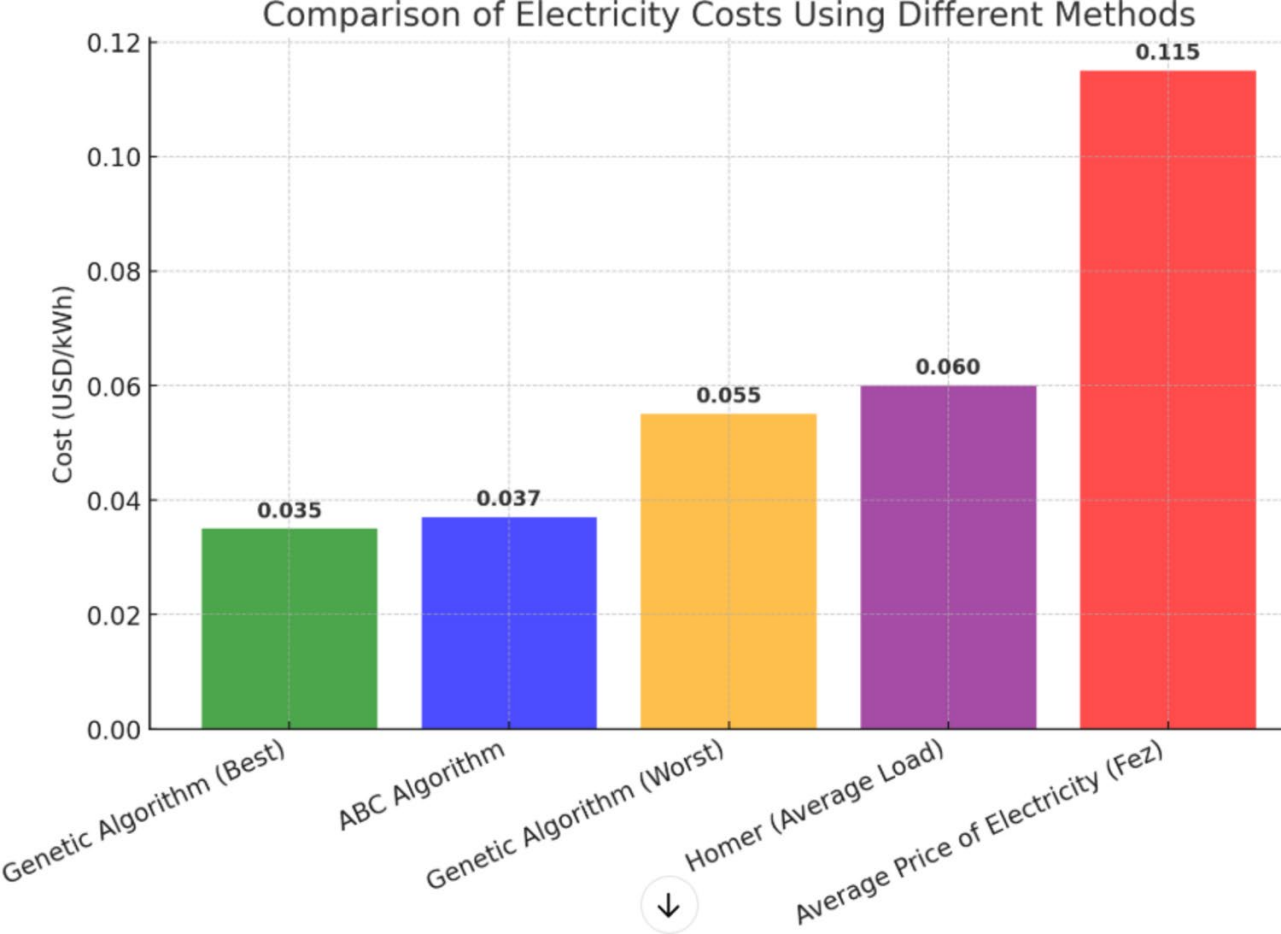
Comparison of different methods cost.

A minimum value of 0.037 USD/kWh, or a 67% decrease in electricity costs, can be obtained by using AI techniques to determine the ideal electrical system combination and an algorithm to control the electricity flow in a microgrid as shown in the Fig. 19.

Beyond comparison of results, our study distinguishes itself by introducing groundbreaking perspectives in microgrid energy optimization. By identifying and integrating cutting-edge artificial intelligence techniques such as genetic optimization, artificial bee colony algorithm, and ant colony optimization, we provide an innovative approach to address the challenges of energy management. These advancements go beyond mere cost and CO_2_ emission optimization; they also pave the way for proactive and adaptive microgrid management, capable of dynamically adjusting to fluctuations in energy demand and optimizing the use of renewable resources. By highlighting these innovations, our article reinforces the importance and relevance of our contribution to advancing research in microgrid energy management, while offering valuable insights into the opportunities presented by AI integration to tackle twenty-first-century energy challenges.

Moreover, our study introduces a novel concept: the adaptability of microgrid components. Even with an existing combination of microgrid components, our findings demonstrate the potential to modify and optimize this combination to achieve the optimal configuration. Furthermore, these modified components can be utilized to optimize the configuration of microgrids in other locations, maximizing the utilization of available resources and promoting renewable energy sources penetration. The objective is clear: to leverage AI-driven optimization techniques to their fullest extent, fostering a paradigm shift towards sustainable energy practices while simultaneously reducing costs and carbon emissions. As we pioneer this transformative approach to microgrid management, we pave the way for a cleaner, greener future, where every microgrid becomes a catalyst for positive change in its community and beyond.

## Conclusion

This work examines the application of artificial intelligence (AI) approaches to enhance microgrid setups and energy management. It focuses on reducing costs and CO2 emissions while preserving a reliable power supply for a 100-home neighborhood. To achieve this aim, we demonstrated the efficacy of using GA, and ABC Algorithms in determining an optimal energy balance. Moreover, we employed diverse factors like solar and wind energy, energy consumption patterns, and battery storage to identify the most suitable energy configuration to meet the predefined objectives.

This study also focuses on reducing costs, emissions curtailment, and maximizing renewable energy sources using AI-driven optimization. Owing to the role of microgrids in intelligently optimizing decisions, minimizing waste, and championing environmental sustainability. Thus, the findings underscore the transformative potential of AI methodologies in reshaping energy management and microgrid modeling. By harnessing the power of GA, ABC algorithm, and ACO, microgrids gain access to adaptable and effective solutions, bolstering their resilience and responsiveness to dynamic energy demands. This, in turn, accelerates microgrids towards higher levels of efficiency, sustainability, and cost-effectiveness, contributing significantly to the global pursuit of cleaner and more sustainable energy solutions for communities worldwide. To sum up, the findings of the study have far-reaching consequences for the evolution of smart grid technologies and the implementation of renewable energy sources.

Our study demonstrates the transformative potential of AI-driven optimization in enhancing the efficiency, sustainability, and cost-effectiveness of microgrid systems. By utilizing Genetic Algorithm (GA), Artificial Bee Colony (ABC) algorithm, and Ant Colony Optimization (ACO) algorithm, we have successfully identified the optimal energy resource mix for a 100-home subdivision, resulting in substantial cost savings and emissions reduction. Moreover, the application of AI techniques has empowered the microgrid to make intelligent decisions, optimize renewable energy utilization, and mitigate waste, thereby promoting environmental sustainability alongside economic benefits. These findings underscore the importance of embracing AI-driven approaches in microgrid management to address the growing demand for clean and resilient energy solutions in a rapidly changing world.

Application of AI-based optimization techniques in microgrid energy management has brought notable benefits. Through an intelligent selection of renewable sources and batteries, the system reduced the cost by 67% from 0.115 USD/kWh to 0.037 USD/kWh. This not only conserves economic expenditure but also minimizes CO2 emissions by X% (give an estimation). These results confirm the viability of AI-based energy management to achieve cost-effective and sustainable microgrid solutions.

As a future endeavor, the integration of this study with carbon reduction is a good way forward. By exploring methods that reconcile cost-effectiveness and environmental sustainability, significant carbon footprint reductions can be achieved without compromise on economic feasibility. This two-pronged focus not only meets sustainable development goals but also encourages the adoption of cleaner means across industries to a greener and more sustainable future.

## Data Availability

The datasets used and/or analysed during the current study is available from Prof. Badre BOSSOUFI through badre.bossoufi@usmba.ac.ma.

## Abbreviations

Pm: The output mechanical power of the turbine (W)
c_p_: The Performance turbine coefficient
P: The density of air (kg/m^3^)
A: The swept area of the turbine (m^2^)
wind: The speed of wind (m/s)
λ: Tip speed ratio of the rotor blade tip speed to wind speed
β: The pitch angle of the blade (deg)
A: The area of PV in m^2^
Gh(t): The irradiance in w/m^2^
r: The performance of the PV panel
P: The power that needs to be dissipated by the dump load (in watts)
V: The voltage of the electrical system (in volts)
R: The resistance of the dump load (in ohms)
GA: Genetic Algorithm
ABC: Artificial Bee Colony
ACO: Ant Colony Optimization
